# Microgravity-like Crystallization of Paramagnetic Species in Strong Magnetic Fields

**DOI:** 10.3390/ijms25105110

**Published:** 2024-05-08

**Authors:** Arkady A. Samsonenko, Natalia A. Artiukhova, Gleb A. Letyagin, Alexey S. Kiryutin, Ivan V. Zhukov, Sergey L. Veber

**Affiliations:** International Tomography Center of the Siberian Branch of the Russian Academy of Sciences, 3a, Institutskaya Str., Novosibirsk 630090, Russia; a.samsonenko@tomo.nsc.ru (A.A.S.); natalya.artyukhova@tomo.nsc.ru (N.A.A.); gl@tomo.nsc.ru (G.A.L.); kalex@tomo.nsc.ru (A.S.K.); i.zhukov@tomo.nsc.ru (I.V.Z.)

**Keywords:** crystal growth, microgravity conditions, paramagnetic crystals, strong magnetic field, levitation

## Abstract

The crystallization of paramagnetic species in a magnetic field gradient under microgravity-like conditions is an area of interest for both fundamental and applied science. In this paper, a setup for the crystallization of paramagnetic species in the magnetic field up to 7 T generated by a superconducting magnet is described. The research includes calculations of the conditions necessary to compensate for the gravitational force for several types of paramagnetic substances using the magnetic field of superconducting magnets (4.7 T, 7 T, 9.4 T, and 16.4 T). Additionally, for the first time, the crystallization of copper sulfate and cobalt sulfate, as well as a mixture of copper sulfate and cobalt sulfate under gravitational force compensation in a superconducting magnet, was performed. This paper experimentally demonstrates the feasibility of growing paramagnetic crystals within the volume of a test tube on the example of copper and cobalt sulfate crystals. A comparison of crystals grown from the solution of a mixture of copper and cobalt sulfates under the same conditions, with and without the presence of a magnetic field, showed changes in both the number and size of crystals.

## 1. Introduction

Crystal growth under microgravity conditions has shown a number of benefits [1,2,3]. Levitation in a magnetic field gradient can be considered as an approximation of microgravity achieved in laboratory conditions. Perhaps the most well-known example is the levitation of a frog in a high magnetic field [4]. Unlike for diamagnetic materials, the steady-state levitation of paramagnetic ones is not possible due to fundamental reasons [5]. Indeed, the condition for a stable equilibrium position of a magnetic species in an external stationary magnetic field is a positive Laplace value of the potential energy in a magnetic field, which is only possible for diamagnetic species. In a magnetic field, in addition to the effect of compensating for the gravitational force, a number of other effects can appear. For instance, the orientation of species in a magnetic field due to anisotropy of magnetic susceptibility and a non-symmetrical shape of the species [6], or an extremely weak thermodynamic effect [6]. Furthermore, a magnetic field has a significant effect on solutions [6,7,8,9]. Several studies have demonstrated the experimental and theoretical potential to reduce convection in a solution within a magnetic field gradient [10,11,12,13,14].

The majority of investigations have focused on the crystal growth in a magnetic field dedicated to diamagnetic crystals [15,16], including protein crystals [17,18]. For paramagnetic compounds, some rare studies have been carried out on their solutions [19,20,21], and in some cases, they have been used as crystallization agents [22,23]. Only a few studies have focused on the growth of paramagnetic crystals in a magnetic field. However, the results of these investigations are inconsistent and fail to explain the observed changes in the growth rate [24,25,26,27]. E. Vlieg and colleagues have emphasized the role of convection during the growth of paramagnetic crystals in the magnetic field gradient, suggesting it can have a significant impact to the crystallization process [11,12,13].

Nowadays, many laboratories possess sources of strong magnetic fields. Perhaps the most straightforward method to produce relatively strong magnetic fields is by using neodymium (NdFeB), ceramic (ferrite), alnico, and samarium cobalt (SmCo) magnets. However, the remanence in such permanent magnets is capped at 1 T or slightly more [28]. In turn, superconducting magnets [29] can produce fields well beyond 20 T, and the majority of the crystal growth investigations have been conducted using these magnets. Even higher constant magnetic fields, exceeding 40 T, can be achieved with composite and resistive magnets [30].

In this study, we consider widely distributed nuclear magnetic resonance (NMR) magnets as a source of strong magnetic fields for crystal growth of paramagnetic compounds on the example of a 300 MHz wide-bore NMR magnet (7 T). We characterized the magnetic field profile of superconducting magnets of Bruker NMR spectrometers with proton frequencies of 200, 300, 400, and 700 MHz. For each of these magnets, we calculated the pull-in magnetic force acting on various types of paramagnetic species. By balancing the pull-in magnetic, buoyant, and gravity forces, we determined the necessary parameters of paramagnetic species to achieve microgravity conditions in different solvents. This study also details the experimental setup used for crystallization in the magnetic field of a superconducting NMR magnet. Specifically, we focus on the crystallization of copper sulfate, cobalt sulfate, and a mixture of copper and cobalt sulfates under gravitational force compensation. We also compare crystals grown both in the presence and absence of a magnetic field.

## 2. Results and Discussion

### 2.1. Theoretical Considerations

The force acting on a small magnetic body (whose dimensions are small compared to the spatial variation of a magnetic field) in an external magnetic field can be written as follows:

(1) $$F_{m}=V\frac{\chi_{b}-\chi_{s}}{{µ}_{0}}\nabla (\frac{B^{2}}{2})$$

where V is the volume of the body, χ_b_ (unitless) is the volume magnetic susceptibility of the body immersed in the solution, χ_s_ (unitless) is the volume magnetic susceptibility of the solution, B is the magnetic flux density, µ_0_ is the vacuum permeability, equal to 1.25663706212 (19) × 10^−6^ N/A^2^., and ∇—vector differential operator.

In addition to the pull-in magnetic force, gravitational and buoyant forces also act on the body. The vertical component of the lifting force per unit mass of the body, in units of free-fall acceleration, can be expressed as follows:

(2) $$f=\frac{\chi_{b}-\chi_{s}}{2g{µ}_{0}\rho_{b}}\frac{d\left(B^{2}\right)}{dz}-\left(1-\frac{\rho_{s}}{\rho_{b}}\right)$$

where $\rho_{b}$ is the density of the paramagnetic crystal, $\rho_{s}$ is the density of the solution in which the crystal is placed, and g is the gravitational acceleration, taken as 9.8 N/kg.

Equation (2) establishes the relationship between the force acting on an object and the intensity of the magnetic field, taking into account the properties of the object and the solution in which it is immersed. Based on the structure of Equation (2), one can deduce that changes in the force acting on an object, due to variations in both the solution density and its paramagnetic susceptibility, might cause a shift in crystal growth position as the solution gets depleted of dissolved compounds. In the following sections of this manuscript and its supplementary material, the term “magnetic field” will pertain to the vector of magnetic flux density B, a convention widely accepted in chemical literature.

### 2.2. Magnetic Field Characterization

The magnetic field distribution in the superconducting magnets of NMR spectrometers (Bruker BioSpin AG, 8117 Fällanden, Switzerland), specifically Bruker 200 MHz (Ultrashield 54 mm), 300 MHz (Spectrospin 89 mm), 400 MHz (Ascend^TM^ Ultrashield 54 mm), and 700 MHz (Ascend^TM^ Ultrashield 54 mm), was characterized. Among these magnets, the Bruker 300 MHz magnet is unshielded and features a larger warm bore with a diameter of 89 mm, while other magnets used here have 54 mm warm bore. The larger warm bore diameter simplifies access to a magnetic field and provides greater flexibility for the insert designed for performing the crystallization experiments. Consequently, all experiments detailed in this manuscript utilized the wide bore magnet of the Bruker 300 MHz NMR spectrometer.

To determine the optimal position of the test tube containing crystals within the magnet during growth, the magnetic field profile within the entire warm bore of the magnet of a NMR spectrometer Bruker 300 MHz was characterized. A paramagnetic body cannot achieve a stable equilibrium state within a magnetic field, leading the crystals to grow on the test tube wall. Nevertheless, at a distance equivalent to the tube radius (r = 7 mm), the lifting force remains relatively unchanged. Therefore, by characterizing the magnetic field along the axis of the magnet, it is possible to estimate the position of crystal growth. A comprehensive magnetic field distribution is provided in Sections B1 and B2 of the Supplementary Materials.

**Figure 1:**
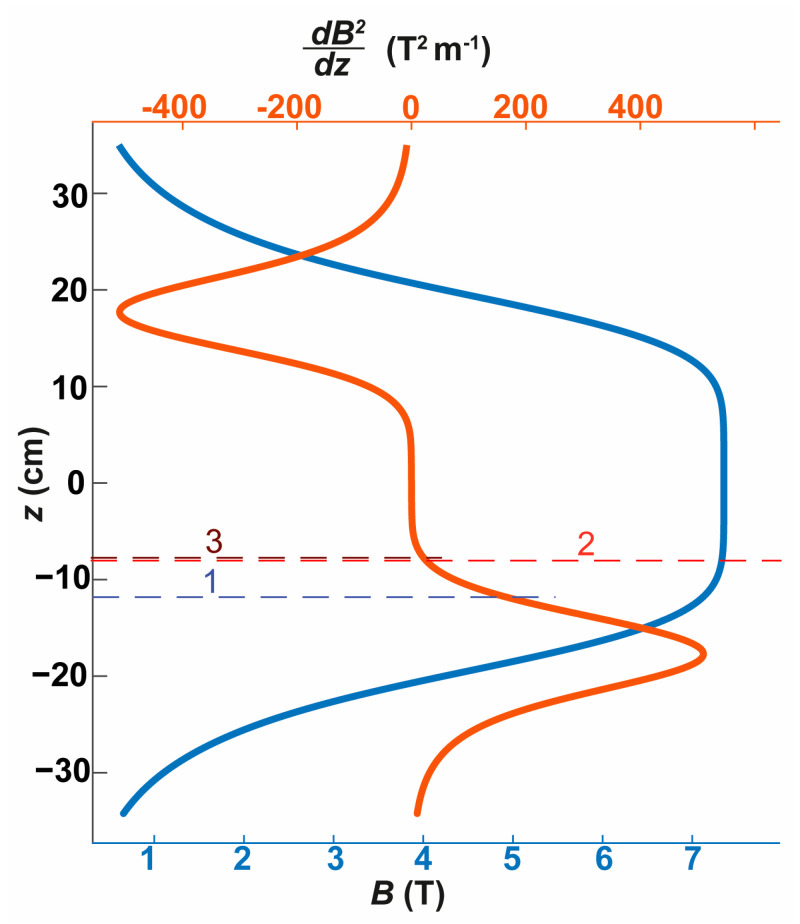
The magnitude of magnetic flux density B and the gradient $\frac{d(B^{2})}{dz}$ along the axis of the magnet of an NMR spectrometer Bruker 300 MHz. The vertical coordinate z is measured from the magnet’s field symmetry plane. Horizontal lines mark the coordinates where the magnetic field gradient and Archimedes force balance out the gravitational force along the magnet’s axis. The positions calculated for CuSO_4_∙5H_2_O, CoSO_4_∙7H_2_O, and FeSO_4_∙7H_2_O are denoted as 1, 2, and 3, respectively.

Figure 1 shows the dependence of the B and $\frac{d(B^{2})}{dz}$ values on the vertical coordinate of the magnet. In addition, the figure illustrates the positions corresponding to the zero projection of the force f (Equation (2)) for the following types of crystals: CuSO_4_∙5H_2_O, CoSO_4_∙7H_2_O, and FeSO_4_∙7H_2_O placed in distillated water. Since the solution itself, from which the crystals grow, is also paramagnetic, the Archimedes force acting on a crystal placed in the solution changes as the solution becomes depleted of dissolved compounds. To estimate this effect, we calculated the positions where gravitational force gets compensated for crystals in saturated solutions at temperatures between 20 °C and 25 °C, and in distillated water. The most significant difference in the position of gravitational force compensation between a crystal in its saturated solution and in distillated water was observed for FeSO_4_∙7H_2_O, with a difference of 4 mm. This indicates that the shift in the position of force compensation during crystal growth is much lower compared to the height of the test tube, and thus, this effect can be excluded from consideration. From the data presented, it is evident that within the 300 MHz NMR magnet, the gravitational force can be compensated for each of the mentioned crystals.

It is noteworthy that the growth positions, which correspond to microgravity conditions, for paramagnetic compounds of differing magnetic susceptibilities can be substantially distinct. For example, there is an estimated 4 cm discrepancy between the growth positions of CuSO_4_∙5H_2_O with a spin 1/2 and CoSO_4_∙7H_2_O with a spin 3/2. This difference suggests that the magnet could theoretically serve as a separator for compounds with notably varied magnetic susceptibilities. Such a separation function could be advantageous in various applications where differentiation of compounds based on their magnetic properties is required. To explore this phenomenon, crystal growth experiments were carried out using a mixture of copper and cobalt sulfates. Experiments with FeSO_4_∙7H_2_O were not conducted in this study. For a more comprehensive analysis of the dependence of the required magnetic field gradient $\frac{d\left(B^{2}\right)}{dz}$ on the characteristics of paramagnetic compounds to balance the gravitational force, refer to Sections B3 and B4 of the Supplementary Materials.

### 2.3. In-Magnet Crystal Growth Procedure

For all experiments described in this article, saturated aqueous solutions of CuSO_4_∙5H_2_O and CoSO_4_∙7H_2_O, as well as an aqueous solution containing a mixture of copper and cobalt sulfates, were prepared at 35 °C. These solutions were then placed in a thermostat set at 25 °C and subsequently cooled down to 10 °C at a rate of 0.6 °C per hour. Seed crystals were introduced in advance for all experiments. Specifically, CuSO_4_∙5H_2_O and CoSO_4_∙7H_2_O crystals were used as seeds for their respective solutions. For experiments with the mixed solution, only a CoSO_4_∙7H_2_O crystal was used as a seed.

Given the design limitations of the insert, simultaneous crystal growth both inside and outside the magnet was not possible. Therefore, crystal growth experiments in the presence and absence of a magnetic field were conducted on different days, but under identical temperature conditions. For experiments focused on crystal growth from a mixed solution of copper and cobalt sulfates, a single aqueous batch was prepared and used for both sets of experiments (with and without a magnetic field).

For the crystal growth experiments using a copper sulfate solution, a total of two trials were conducted: one in the presence of a magnetic field and one without. For cobalt sulfate, four experiments were conducted within a magnetic field, while two were carried out without it. Regarding the mixed solution of copper and cobalt sulfates, three experiments each were performed both with and without a magnetic field.

Without a magnetic field, crystals predominantly formed at the bottom of the test tube, leading to a reduced effective growth area compared to growth area within the magnetic field gradient. To counteract the potential effects of this reduced area, and to differentiate it from the influence of the magnetic field itself, the test tube was tilted to a horizontal position during growth outside the magnet in the experiments involving the growth from a mixed solution of copper and sulfate crystals. This orientation also facilitated easier extraction of the crystals for subsequent analysis, reducing the risk of crystal damage. Conversely, in experiments involving CuSO_4_∙5H_2_O and CoSO_4_∙7H_2_O separate solutions, where the primary objective was to demonstrate gravitational force compensation in the magnetic field, crystals were grown in the vertically oriented test tube outside the magnet.

For all experiments conducted in a magnetic field, the tube’s positioning was consistent: the tube bottom was located 16 cm below the magnet center (refer to Figure 1).

### 2.4. Growth of CuSO_4_∙5H_2_O Crystals

**Figure 2:**
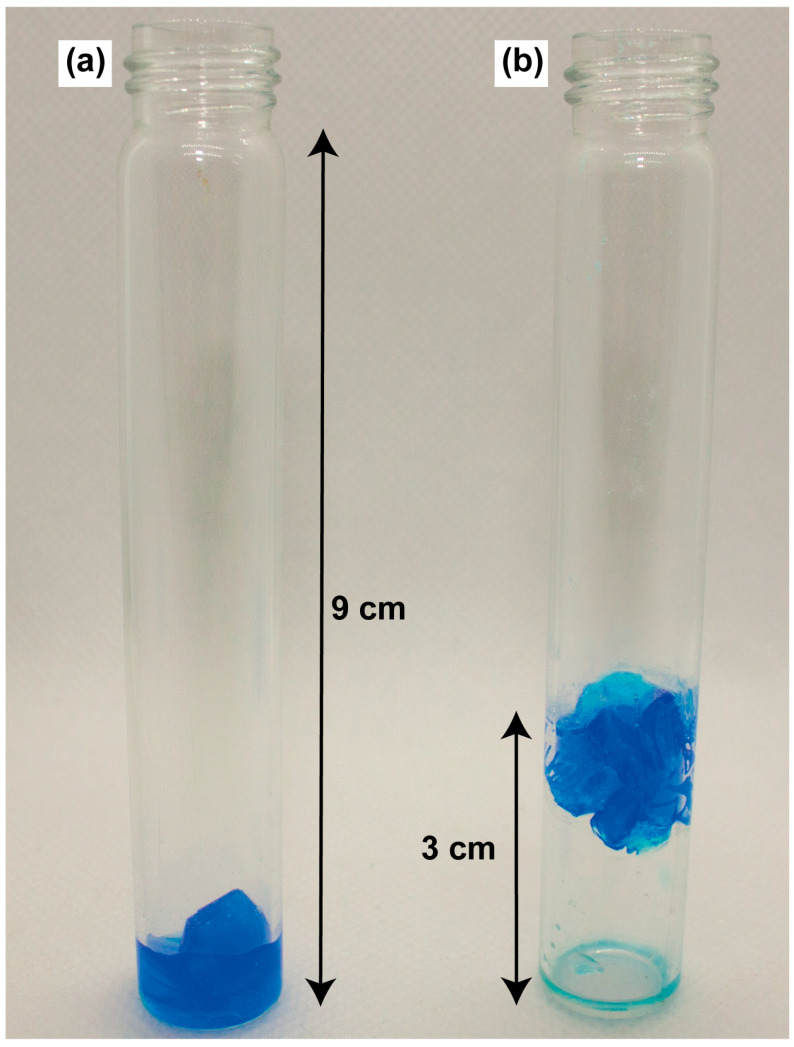
Photograph of the CuSO_4_∙5H_2_O crystals grown without a magnetic field (**a**) and in a magnetic field gradient (**b**).

The crystallization of CuSO_4_∙5H_2_O was carried out from their saturated aqueous solution obtained at 35 °C with the use of a seed crystal. Two separate experiments were conducted to grow CuSO_4_∙5H_2_O crystals: one without any magnetic field influence and another in the presence of a magnetic field gradient. Figure 2 captures the grown crystals.

These experiments validate the feasibility of paramagnetic crystal growth within the volume of a test tube under the influence of a magnetic field. It is worth noting that during growth in a magnetic field, despite the presence of residual horizontal forces, their magnitude is significantly less than the gravity force (see in Section B of the Supplementary Materials). There was no observable difference in the shape, size, or quantity of crystals formed under the influence of a magnetic field gradient compared to those grown without it. Analogous outcomes were noted for CoSO_4_∙7H_2_O crystals, with more details provided in Section D of the Supplementary Materials.

### 2.5. Growth from a Solution of a Mixture of Copper and Cobalt Sulfates

**Figure 3:**
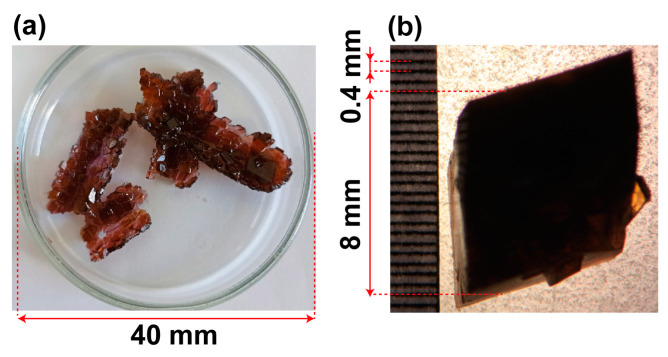
The crystals that were grown from a solution of a mixture of Cu and Co sulfates in two different conditions: without (**a**) and with (**b**) a magnetic field.

The experiments conducted on crystallization from a solution of a mixture of copper and cobalt sulfates, in which cobalt and copper are in a 1:1 molar ratio, revealed distinct differences between the crystals grown with and without a magnetic field. Figure 3 showcases this contrast, where (a) displays crystals grown in the absence of a magnetic field, and (b) shows crystals formed in the magnet (see Section E of the Supplementary Materials for other photos). Notably, when grown from the same solution and under identical temperature conditions, there was a significant discrepancy in both the number and size of crystals obtained in the two scenarios. In the presence of a magnetic field, only a single large crystal (8–12 mm) was observed, while the same solution yielded a cluster of relatively small crystals (1–3 mm) when no magnetic field was applied.

It is known that for Cu-substituted bieberite (Co_1−x_Cu_x_)SO_4_∙7H_2_O, a solid solution forms when the copper content is within the range of 0 ≤ x ≤ 0.46. Conversely, in chalcanthite CuSO_4_∙5H_2_O, only up to 3% of the metal sites can be substituted by Co^2+^ ions [31]. Given that our study focuses on a 1:1 Cu:Co solution, which contains copper just beyond the threshold for bieberite-like solid solution formation, we anticipated the crystallization of both substituted bieberite (Co_1−x_Cu_x_)SO_4_∙7H_2_O and chalcanthite CuSO_4_∙5H_2_O from excess copper sulfate. These two phases are distinct not only in their water content, but also in their crystal systems and symmetries—the heptahydrate is monoclinic ($P2_{1}/c$ space group), while the pentahydrate is triclinic ($P\overset{-}{1}$). Furthermore, Redhammer et al. [31] have demonstrated that the bond lengths in the coordination environments of (Co_1−x_Cu_x_)SO_4_∙7H_2_O metal centers are affected by the degree of substitution *x*. Therefore, single-crystal X-ray diffraction (SC XRD) serves as a robust tool not only for distinguishing between the heptahydrate and pentahydrate phases, but also for confirming the formation of a solid solution.

SC XRD was used to characterize two single crystals, selected from batches grown with and without an external magnetic field, respectively. It is important to note that the crystals analyzed were obtained from the same original solution, which was divided into equal parts, with a time difference of one day between crystal growth. X-ray diffraction analysis shows the formation of Cu_x_Co_1−x_SO_4_∙7H_2_O in both cases with similar bond lengths in the metal center environment characteristic of solid solution with x~0.46 (Table 1). The Jahn–Teller elongated bond length M-O (which is most sensitive to the degree of substitution) is 2.3107(13) Å and 2.3259(18) Å for crystals grown in the magnet and outside of it, respectively. These values are close to the literature data [31] for Cu_x_Co_1−x_SO_4_∙7H_2_O with copper content x of 0.46: the difference between respective bond lengths is less than 0.03 Å (see Table 1).

UV–visible spectrophotometric analysis was conducted on the crystalline samples obtained from all experiments involving the growth of crystals from a solution of mixture of copper and cobalt sulfates. The results did not reveal any significant quantitative differences in the Cu and Co content when comparing samples grown in the presence of a magnetic field with those grown in its absence. For the formula Cu_x_Co_1−x_SO_4_∙7H_2_O, the analysis indicated that the value of x ranges from 0.36 to 0.41.

For elemental analysis, samples from each batch of crystals grown with and without a magnetic field were examined. Notable variations in the Cu and Co content among the different crystals were observed, obtained both in the magnetic field and in the absence of it; nonetheless, these variations were consistent with published data on Cu content in bieberite-like structures. Elemental analysis confirmed that for the compound Cu_x_Co_1−x_SO_4_∙7H_2_O, the value of x in the crystals ranged from 0.34 to 0.4. These results, in conjunction with those from single-crystal X-ray diffraction (SC XRD) and spectrophotometry, confirm the formation of a Cu_x_Co_1__−x_SO_4_∙7H_2_O solid solution.

Differences in the crystallization outcomes from an aqueous solution of a mixture of copper and cobalt sulfates can be caused by several possible factors:

(1) When the magnetic field gradient balances out the gravitational force, magnetic dipole interactions between crystallization centers can induce crystal drift. The drift phenomenon can take place at a rate that exceeds the thermal rate and is observed in crystals with a volume exceeding (0.04 mm^3^), located at a distance roughly equivalent to the diameter of the test tubes utilized (1 cm). Depending on the relative position of crystals, the dipole force can lead to either repulsion or attraction of the crystals. However, attraction occurs more frequently than repulsion, causing the crystal centers to stick together and form a single center. Considering that the final size of the developed crystals significantly exceeds 0.1 mm, it is likely that this attraction and subsequent merging due to dipole forces plays a substantial role in the observed crystal growth patterns (detailed analysis is provided in Section G1 of the Supplementary Materials).
(2) When crystals are situated in the magnetic field of an NMR magnet, they are subjected to both axial force and radial forces. Assuming that the test tube in practical experiments has some off-center displacement from the axis of the magnet, it results in force acting along the perimeter of the tube. Consequently, this creates a “pocket” where the crystals are collected and aggregate, potentially influencing the overall crystal growth (refer to Section G2 of the Supplementary Materials for more details).
(3) During crystal growth, a zone depleted of dissolved species forms around the crystal, leading to a slight reduction in the solution’s density in this vicinity. In the absence of a magnetic field, this density gradient leads to the initiation of natural convection, similar to the convection observed in a heated liquid. In the presence of a magnetic field, the convection can be significantly suppressed due to the appearance of a force that compensates for gravitational force. This phenomenon has been partially studied and is supported by evidence in the literature [11,12,13,32]. The suppression of convection near the growing crystal means it grows in a solution with a lower local concentration compared to the bulk solution. This change in the local concentration impacts the rate of the nucleation process. With the reduced concentration, the likelihood of new crystal nucleation decreases, leading to a slower nucleation rate [33]. As a result, rather than the formation of numerous small crystals in proximity of a seed, the growth conditions favor the development of a single large crystal. Thus, the suppression of convection can play a crucial role in promoting the growth of a predominant crystal, significantly affecting the final size of the crystal (for additional details, refer to Section G3 of the Supplementary Materials).

## 3. Materials and Methods

### 3.1. Superconducting Magnet

The axial magnetic field of the Bruker 300 MHz NMR spectrometer was measured with a resolution of 2 mm on the central axis of the magnet and at a radial distance of 40 mm from this axis with a Hall sensor of a Lakeshore 475 DSP gaussmeter. Given that potential magnetic field distributions are constrained by Maxwell’s equations, such measurements enable the estimation of both the radial and the axial component of the magnetic fields throughout the warm bore (detailed in Section A of the Supplementary Materials). The field characteristics for the entire warm bore of the 300 MHz magnet, along with the field characterizations on the axis of the 200 MHz, 400 MHz, and 700 MHz magnets, are presented in Sections B1 and B2 of the Supplementary Materials.

### 3.2. Experimental Setup

**Figure 4:**
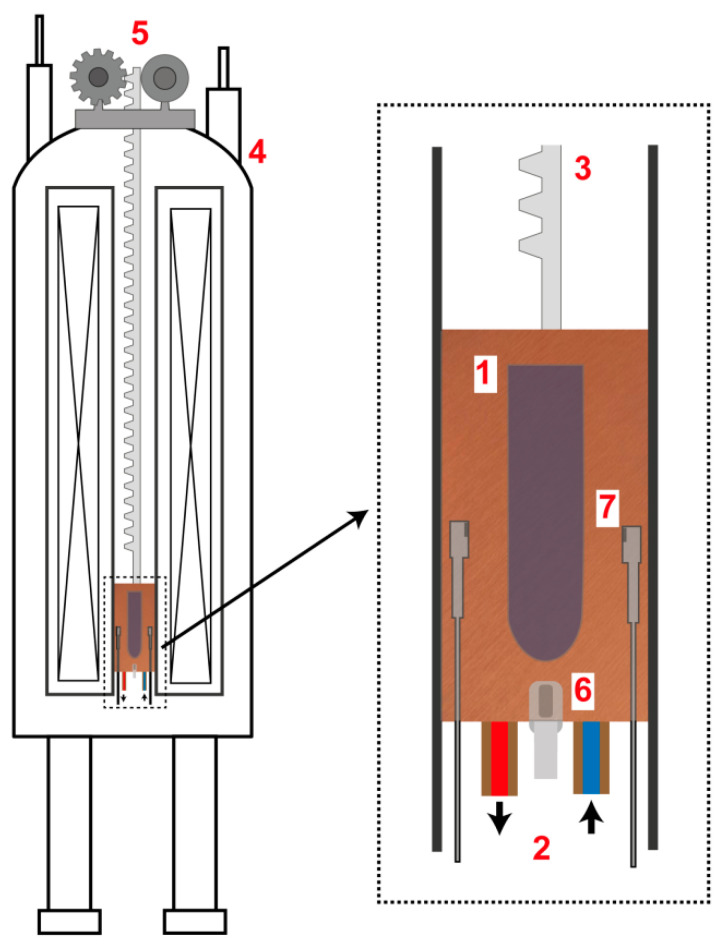
Insert for crystal growth within the NMR magnet. (1) Temperature-regulated chamber for the sample; (2) coolant circulation in the thermostat, with the arrows showing the coolant flow direction; (3) rail for precise insert positioning; (4) NMR magnet; (5) positioning system powered by a stepper motor; (6) temperature sensor; (7) video probes.

To conduct the experiments with precise control over the temperature during crystal growth, a custom insert was designed specifically for the 300 MHz NMR cryomagnet. This insert is depicted in Figure 4. The sample (1) was placed into a temperature-regulated chamber (thermostat) within the insert. To manage the internal temperature, a coolant (2) was circulated within this insert through the channels and its temperature was stabilized. To enable the movement of the rail (3) connected to the insert along the magnet warm bore (4), a positioning system (5) driven by a step motor was utilized. The thermostat temperature was consistently monitored using a sensor (6). Additionally, the insert was equipped with video probes (7) to allow direct visualization of the samples inside.

The thermostat can regulate temperatures ranging from room temperature down to 10 °C, following a specified cooling rate. The experiments focusing on the growth of CuSO_4_∙5H_2_O and CoSO_4_∙7H_2_O crystals from their solutions, as well as from a combined solution of copper and cobalt sulfates, were conducted within this system. Detailed technical specifications of the insert can be found in Section C of the Supplementary Materials.

While the video probes provide real-time monitoring of crystal positioning during growth, they have a limitation. They contain magnetizable components, which can alter the magnetic field distribution around the sample. Due to this, the video probes were only used in initial test runs. Videos from these preliminary experiments are available in Section G of the Supplementary Materials.

### 3.3. Analysis Methods

The crystals obtained from the CuSO_4_∙5H_2_O and CoSO_4_∙7H_2_O solutions were photographed. For crystals derived from the mixed solution of sulfates, a number of additional characterizations were carried out by employing methods such as X-ray diffraction analysis, UV–visible spectrophotometry, and elemental analysis.

#### 3.3.1. UV–Visible Spectrophotometry

**Figure 5:**
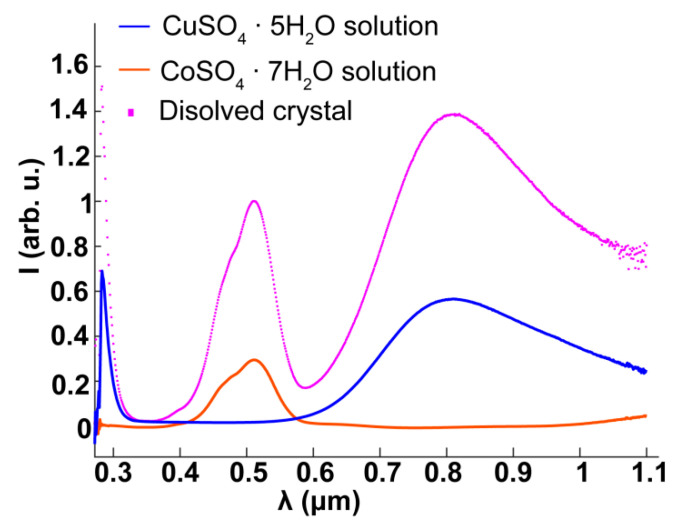
Spectrum of CuSO_4_∙5H_2_O and CoSO_4_∙7H_2_O water solutions compared with spectra from a dissolved crystalline sample grown from a mixed solution of copper and cobalt sulfates.

For the UV–visible spectrophotometry characterization, the grown crystals were dissolved in distilled water at an approximate concentration of 20 mg/mL. As depicted in Figure 5, one of the recorded spectra is showcased and compared with the spectra of CuSO_4_∙5H_2_O and CoSO_4_∙7H_2_O solutions. The distinct spectral differences between the spectra of copper and cobalt sulfate solutions provide a basis for estimating the Cu and Co content in the crystalline samples derived from aqueous solution containing both copper and cobalt sulfates.

#### 3.3.2. Single-Crystal X-ray Diffraction

Single-crystal X-ray diffraction experiments were carried out using a Bruker AXS diffractometer SMART APEX II (Mo Kα radiation). Data were collected using Apex2 [34], and the intensity data were corrected for absorption using multi-scan techniques (SADABS program, version 2.10) [35]. The structures were solved by direct methods and refined by full-matrix least-squares in an anisotropic approximation for all non-hydrogen atoms. The H atoms were located in the difference Fourier map and their coordinates were free to refine during least-square procedure. All calculations on structure solution and refinement were performed with SHELXL 2018/3 software programs [36,37]. Comprehensive crystallographic data are presented in Section H tables of the Supplementary Materials. The obtained Cu_x_Co_1−x_SO_4_∙7H_2_O solid solution crystal structures were uploaded to the joint CCDC/FIZ Karlsruhe deposition service. The numbers are 2,339,711 and 2,339,712 for crystals grown in strong magnetic fields and in ambient conditions, respectively.

#### 3.3.3. Elemental Analysis

Elemental analyses were conducted using an EA-3000 EuroVector analyzer (HEKAtech, Milano, Italy), placed at the Novosibirsk Institute of Organic Chemistry SB RAS. To determine the quantitative composition of Co and Cu ions in the resultant crystals, samples from both magnetically-influenced growth and standard growth (without a magnetic field) were chosen for analysis.

## 4. Conclusions

This research successfully shows the theoretical potential for growing paramagnetic crystals at the microgravity conditions within the volume of a test tube in Bruker NMR magnets with frequencies of 200, 300, 400, and 700 MHz, and also provides a more comprehensive analysis of the field distribution in a 300 MHz magnet. It was revealed that the change in magnetic force resulting from the depletion of the solution by dissolved substances during crystal growth is negligible. Furthermore, this study highlights the theoretical potential for spatially segregating crystals based on their magnetic susceptibility within a magnetic field. Such a separation function could be advantageous in various applications where differentiation of compounds based on their magnetic properties is required.

Experimental investigations conducted on copper sulfate and cobalt sulfate solutions confirmed the feasibility of growing crystals within the volume of a test tube. Nevertheless, there were no significant qualitative differences noted in the shape or quantity of crystals. An in-depth investigation of the crystal growth from a mixed solution of copper and cobalt sulfates indicated the formation of Cu_x_Co_1−x_SO_4_∙7H_2_O crystals under both conditions—with and without a magnetic field. The crystallization under microgravity conditions resulted in the formation of a single large crystal, whereas the absence of a magnetic field led to multiple nucleations. SC XRD proved the differences in the bond lengths for Cu_x_Co_1−x_SO_4_∙7H_2_O crystals, grown with and without an external magnetic field, are minor. UV–visible spectrophotometric and elemental analysis also did not reveal any significant variation in the Cu and Co content in the formed crystals. Possible reasons for observed differences in crystal dimensions are discussed.

Wide availability of superconducting magnets as an internal part of NMR spectrometers, which provide a strong magnetic field, offers researchers in the fields of chemistry and physics opportunities to study the impact of such fields on crystallization processes. The crystallization processes under microgravity conditions impose additional effects and are of interest from both fundamental and practical perspectives. In the case of paramagnetic compounds, the magnetic force can compensate for the vertical gravitational force but introduces a horizontal component, which directs the compound toward the lateral surfaces where the magnetic species ultimately accumulate. The resultant force can be substantially less than the gravitational force, thus approximating the crystal growth conditions to those of microgravity. As demonstrated, microgravity-like conditions are practically achievable for almost any paramagnetic compound using superconducting magnets in NMR spectrometers. These circumstances open up valuable prospects for conducting extensive research into the effects of a magnetic field on crystallization processes, both generally and specifically under microgravity conditions.

## Figures and Tables

**Table 1:** Comparison of selected bond lengths (Å) for Cu_x_Co_1__−x_SO_4_∙7H_2_O crystallized in the present and absent of a magnetic field, in relation to the literature values.

x (Content of Cu in Cu_x_Co_1__−x_SO_4_∙7H_2_O)	~0.4	~0.4	0.46 [31]
Magnetic field, T	~7	Ambient conditions	Ambient conditions
M1–O1	2.0187(12)	2.0229(16)	2.017(1)
M1–O2	2.0965(13)	2.0988(18)	2.099(1)
M1–O3	2.1256(12)	2.1206(16)	2.124(1)
M2–O4	1.9927(11)	1.9859(14)	2.001(1)
M2–O5	1.9972(11)	1.9894(16)	2.003(1)
M2–O6	2.3107(13)	2.3259(18)	2.296(1)

## Data Availability

The data presented in this study are available on request from the corresponding author.

## Supplementary Materials

The following supporting information can be downloaded at: https://www.mdpi.com/article/10.3390/ijms25105110/s1, References [11,31,32,38,39,40,41,42] are cited in the Supplementary Materials.
