# Supplementary material for: Microgravity-like Crystallization of Paramagnetic Species in Strong Magnetic Fields
Source: Int J Mol Sci. 2024 May 8;25(10):5110. doi: 10.3390/ijms25105110 (PMC11120673; doi:10.3390/ijms25105110)
Supplement: Supplementary file 1 [file ijms-25-05110-s001.zip › Supplementary_Materials.pdf]

Supplementary Materials

Microgravity-like crystallization of paramagnetic species in strong magnetic fields.

Arkady A. Samsonenko , Natalia A. Artiukhova , Gleb A. Letyagin , Alexey S. Kiryutin , Ivan V. Zhukov , Sergey L. Veber *

International Tomography Center of the Siberian Branch of the Russian Academy of Sciences, 3a, Institutskaya Str., Novosibirsk 630090, Russia; a.samsonenko@tomo.nsc.ru (A.A.S.); natalya.artyukhova@tomo.nsc.ru (N.A.A.); gl@tomo.nsc.ru (G.A.L.); kalex@tomo.nsc.ru (A.S.K.); i.zhukov@tomo.nsc.ru (I.V.Z.)

* Corresponding author: sergey.veber@tomo.nsc.ru

Contents

A. The magnetic field distribution in the warm bore of the magnet: theoretical consideration S1

B. Detailed characteristics of magnetic field in magnets S2

B1. Characterization of the magnetic field of the unshielded 300 MHz NMR magnet S2

B2. Comparison of the magnetic field profile of superconducting magnets of NMR spectrometers 300, 400, 500, 700 MHz and permanent magnets S3

B3. The conditions for compensating gravitational force in the case of paramagnetic species vary with their magnetic susceptibility and the characteristic $\frac{d(B)^2}{dz}$ of the magnet S6

B4. The conditions for compensating gravitational force in the case of paramagnetic species vary with their molar mass and the characteristic $\frac{d(B)^2}{dz}$ of the magnet S7

C. The design of the insert for crystallization in the magnet of the 300 MHz NMR spectrometer S9

D. Characteristics of the size and number of grown $CoSO_4 \cdot 7H_2O$ crystals S12

E. Detailed characterization of crystals grown from a solution of a mixture of copper and cobalt sulfates. S15

F. The description of video materials S17

G. Analysis of the influence of various effects on crystal growth in the magnetic field S18

G1. Estimation of magnetic dipole force influence S18

G2. Estimation of test tube off-axis displacement effect on possible aggregation of crystal centers S18

G3. Estimation of convection suppression effect S19

H. Crystallographic characteristics and crystallization conditions of $Cu_xCo_{1-x}SO_4 \cdot 7H_2O$ S20

I. References S20

A. The magnetic field distribution in the warm bore of the magnet: theoretical consideration

To accurately estimate the position of crystal growth, a comprehensive understanding of the magnetic field distribution throughout the magnet is crucial. While the z-component of the magnetic field can be measured with high accuracy, measuring the radial component poses challenges. This difficulty arises from the requirement of precisely aligning the orientation of the hall sensor of the measuring instrument, as any deviation can introduce significant measurement errors caused by the influence of a large z-component.

However, despite these challenges, the limited range of possible magnetic field distributions, as governed by the Maxwell equations, allows for the “reconstruction” of the magnetic field based on given boundary conditions.

It follows from Maxwell's equations that:

$$div\boldsymbol{B} = 0 \quad (1S)$$

Since there are no currents in the warm bore of the magnet:

$$rot\boldsymbol{B} = 0 \quad (2S)$$

In a simply connected domain, this implies that:

$$\boldsymbol{B} = -\nabla\varphi \quad (3S)$$

It follows from the (1S), (3S) equations and axisymmetric of a magnet that:

$$\Delta\varphi\,(\mathrm{r},\mathrm{z}) = 0 \quad (4S)$$

Since the vertical field component in the considered magnet of NMR spectrometer Bruker 300 MHz is much larger than the radial field component, the measurement of the radial field component will have a significant error. As a result, measurements were limited to the vertical component of the magnetic field, which is aligned with the magnet axis. These measurements were conducted along the central axis and at a radial distance of R = 40 mm from it.

The results taken at a specific radial distance yield boundary conditions:

$$\frac{\partial\varphi}{\partial z}|_{r=R} = f(z) \quad (5S)$$

It is convenient to take the Fourier transform along the z coordinate and solve the remaining equation with respect to the variable. Then equations (4S) and (5S) are rewritten in the form:

$$\frac{\partial^2\tilde{\varphi}}{\partial r^2} + \frac{\partial\tilde{\varphi}}{r\partial r} - k^2\tilde{\varphi} = 0 \quad (6S)$$

And

$$\tilde{\varphi}|_{r=R} = \frac{\tilde{f}(k)}{ik} \quad (7S)$$

where $\tilde{\varphi}(k,r)$ is Fourier image of $\varphi(z,r)$ and $\tilde{f}(k)$ is Fourier image of $f(z)$:

$$\tilde{f}(k) = \frac{1}{2\pi}\cdot\int_{-\infty}^{\infty} f\, e^{-ikz}dz \quad (8S)$$

Equation (6S) is a modified Bessel differential equation. Since the $\tilde{\varphi}$ function satisfies the condition $\frac{\partial\tilde{\varphi}}{\partial r}|_{r=0} = 0$, the solution to equation (6S) is proportional to the modified Bessel function of the first kind $\mathrm{I}_0(\mathrm{kr})$. Then it possible to write:

$$\tilde{\varphi} = C(k)\cdot I_0(kr) \quad (9S)$$

Where the constant C can be found from Equation (7S):

$$C(k) = \frac{\tilde{f}(k)}{ikI_0(kR)} \quad (10S)$$

As a result:

$$\varphi = \int_{-\infty}^{\infty}\frac{\tilde{f}(k)\cdot I_0(kr)e^{ikz}}{ikI_0(kR)}dk \quad (11S)$$

We are interested in fields:

$$Br = \int_{-\infty}^{\infty}\frac{\tilde{f}(k)\cdot I_1(kr)e^{ikz}}{iI_0(kR)}\,dk \quad (12S)$$

$$Bz = -\int_{-\infty}^{\infty}\frac{\tilde{f}(k)\cdot I_0(kr)e^{ikz}}{I_0(kR)}\,dk \quad (13S)$$

Modified Bessel function of the first kind $I_m(kr)$ has asymptotic behavior at $\pm\infty$ as following[1]:

$$I_m(kr) \propto \frac{e^{|kr|}}{\sqrt{2\pi|kr|}}(1 + O(\frac{1}{kr})) \quad (14S)$$

Because of it, the terms $\frac{I_0(kr)}{I_0(kR)}$ and $\frac{I_1(kr)}{I_0(kR)}$ included in the integrals (12S) and (13S) remain finite throughout the entire domain of definition of the argument and decrease at $\pm\infty$. Thus, the integrals (12S) and (13S) converges for r < R.

Thus, the measurement of the z-component of the magnetic field at some boundary of a cylinder of radius R makes it possible to completely restore the field in the cylinder (a warm bore of the magnet).

B. Detailed characteristics of magnetic field in magnets

B1. Characterization of the magnetic field of the unshielded 300 MHz NMR magnet

Since the magnetic force depends on the $\nabla(\frac{B^2}{2})$ as shown in formula (1), it becomes crucial to assess the distribution of $\frac{d(B)^2}{dz}$ and $\frac{d(B)^2}{dr}$ projections for this vector. The figure 1S (a) shows the results of measuring the magnitude of magnetic field B and the calculated value of $\frac{d(B)^2}{dz}$ on the axis of the Bruker 300 MHz NMR spectrometer (unshielded), which were obtained using measurement with a LakeShore gaussmeter (Lakeshore 475 DSP) with axial Hall sensor HMMA-1808-VR-03. The figure 1S (b) displays the distribution of the field in the entire warm bore, which was achieved by solving the Maxwell equation with boundary conditions at a radius of 40 mm (for more details, refer to the section A). Additionally, using the magnitude of the magnetic field in the warm bore, the distributions of $\frac{d(B)^2}{dz}$ and $\frac{d(B)^2}{dr}$ were determined and plotted on the figures (c) and (d), respectively.

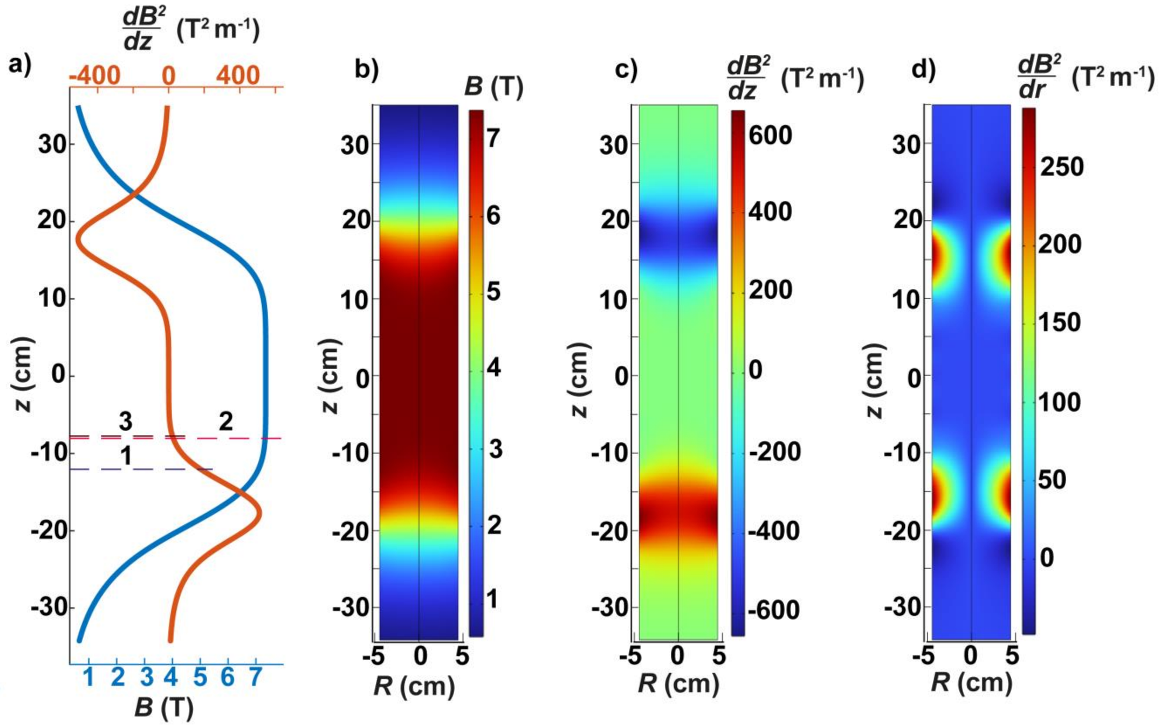

Figure 1S. The field characteristics of the Bruker 300 MHz spectrometer superconducting magnet. (a) The magnitude of field B and value $\frac{d(B)^2}{dz}$ on the axis. z represents the vertical coordinate along the magnet axis, with the zero point selected at the symmetry position of the graph of magnetic field, where it is uniform; (b) The distribution of magnetic field B in the warm bore; (c) the distribution of value $\frac{d(B)^2}{dz}$; (d) the distribution of value $\frac{d(B)^2}{dr}$.

As stated earlier, a paramagnetic species placed in the static magnetic field of arbitrary configuration can not be in a state of stable equilibrium. However, when paramagnetic species are located in the warm bore of the NMR magnet, the radial projection of magnetic force $F_r \sim \frac{d(B)^2}{dr}$ is not big in comparison with the gravity force. The value $\frac{d(B)^2}{dr}$ for the magnet of Bruker 300 MHz spectrometer does not exceed 80 T^2m^{-1} for a test tube with a diameter of 1 cm placed on the central axis, which is almost an order of magnitude less than the maximum value $\frac{d(B)^2}{dz}$.

It is important to note that the value of $\frac{d(B)^2}{dz}$ varies with the radius of the test tube. The maximum difference in this value between the radius of 1 cm and the axis is approximately achieved at the coordinate z, where the value $\frac{d(B)^2}{dz}$ is maximized (~ -16 cm), and this difference is about 5 $T^2 \cdot m^{-1}$. It is two orders of magnitude less than the maximum of value $\frac{d(B)^2}{dz}$. Consequently, the change in lifting magnetic force concerning the radius of the test tube is negligible.

B2. Comparison of the magnetic field profile of superconducting magnets of NMR spectrometers 300, 400, 500, 700 MHz and permanent magnets

The magnetic characteristics of 200, 300, 400, and 700 MHz magnets were compared with the simulated field distribution of the reference permanent magnets, which are ring magnets made of NdFeB grade N30Th. The first permanent magnet has the outer diameter of 120 mm and the inner bore diameter of 20 mm and the length of 100 mm while the second permanent magnet has the outer diameter of 320 mm, the inner bore diameter of 40 mm and the length of 500 mm. These two permanent magnets are vertically magnetized (along the magnet axis), and their remnant flux density is 1.11T, with a recoil permeability of 1.05. Figure 2S and figure 3S show the magnitude of magnetic field B and value $\frac{d(B)^2}{dz}$ on the axis (a) of permanent magnet and magnetic field distribution in the volume for permanent magnets with outer diameter 120 mm (figure 2S) and 320 mm (figure 3S).

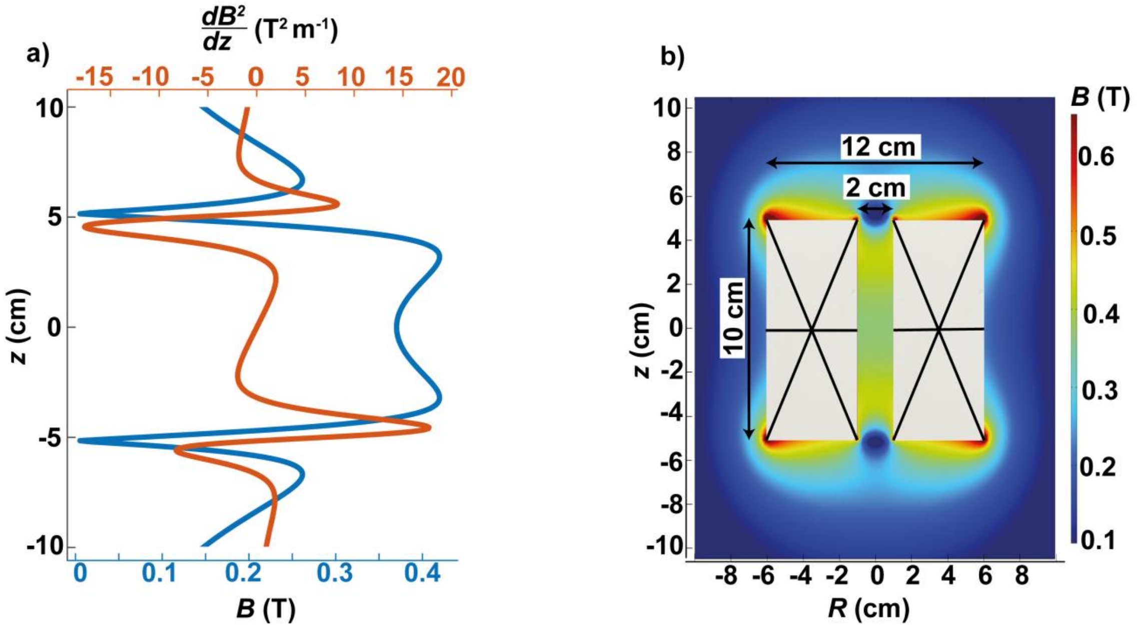

Figure 2S. The field characteristics of a permanent magnet that is vertically magnetized along the magnet axis and has an outer diameter of 120 mm. (a) The magnitude of magnetic field B and value $\frac{d(B)^2}{dz}$ on the axis of the magnet; (b) the distribution of magnetic field B in the volume of the magnet.

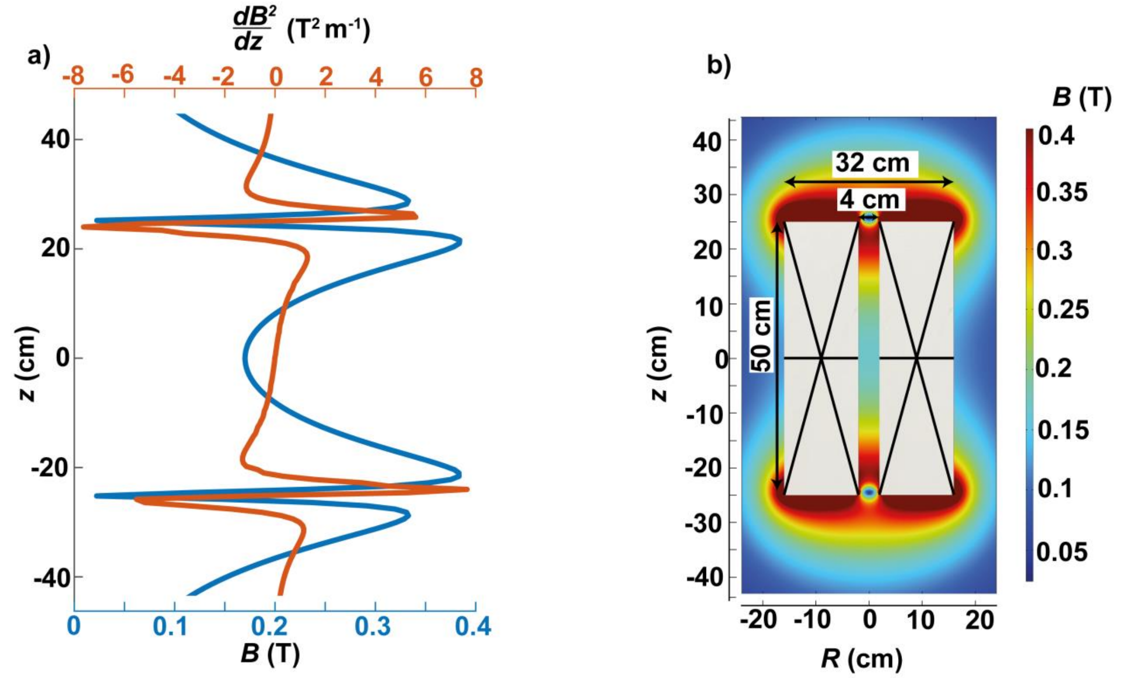

Figure 3S. The field characteristics of a permanent magnet that is vertically magnetized along the magnet axis and has an outer diameter of 320 mm. (a) The magnitude of magnetic field magnitude B and value $\frac{d(B)^2}{dz}$ on the axis of magnet; (b) the distribution of magnetic field B in the volume of magnet.

The third type of permanent magnet is Halbach magnet[2]. This construction allows to get high magnetic field in air gap and actively used for benchtop NMR spectrometers[3]. The magnetization of this magnet changes according following formula:

$$\boldsymbol{M} = Mr(\boldsymbol{e}_\rho cos(\varphi - \pi/2) + \boldsymbol{e}_\varphi sin(\varphi - \pi/2)) \qquad (15S)$$

Where Mr is remanence, $\boldsymbol{e}_\rho$ and $\boldsymbol{e}_\varphi$ are basis vectors. Similarly, this magnet has a cylindrical shape and outer diameter of 120 mm, the inner bore diameter of 20 mm and the length of 100 mm. Figure 4S (a) depicts the magnetization vector field distribution in the horizontal cross section perpendicular to the magnet axis. Figure 4S (b) shows the calculated magnitude of magnetic field B and value $\frac{d(B)^2}{dz}$ on the axis of magnet. The distribution of magnetic field magnitude B in the volume of the magnet is shown of figure 4S (c).

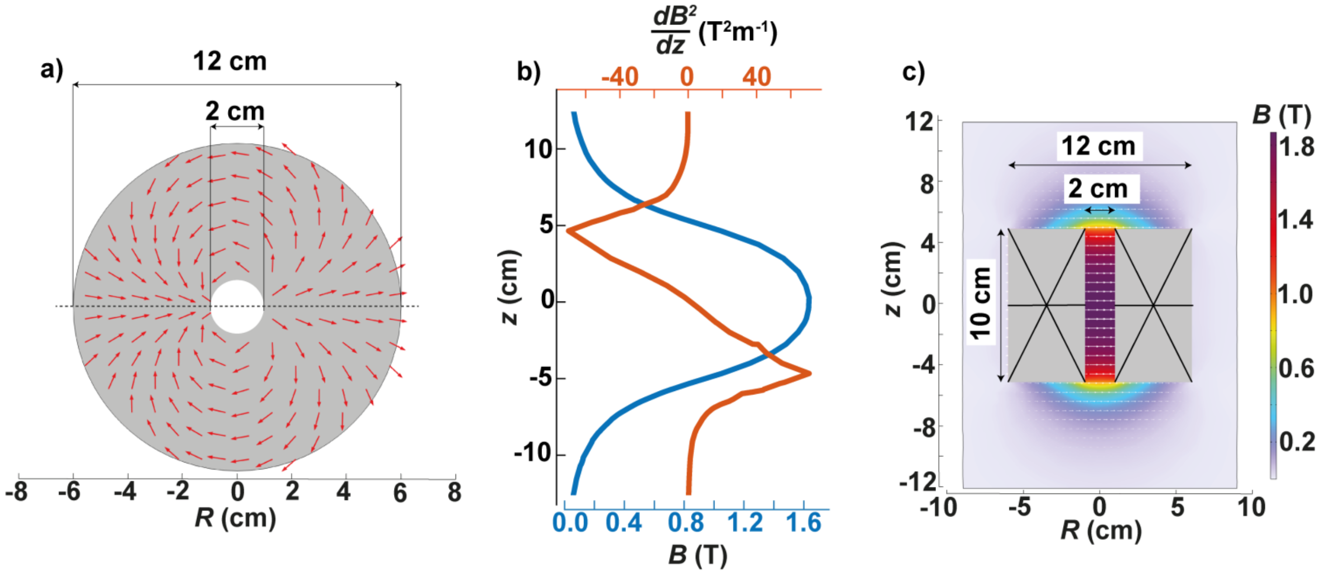

Figure 4S. The field characteristics of the Halbach magnet with outer diameter 120 mm. (a) Magnetization vector field distribution in the horizontal cross section perpendicular to the magnet axis; (b) the magnitude of magnetic field B and value $\frac{d(B)^2}{dz}$ on the axis of magnet; (c) the distribution of magnetic field magnitude B in the volume of the magnet, the section intersects with the dashed line (--) in fig. (a).

Considering that the Halbach magnet lacks axial symmetry, it is beneficial to also analyze the distribution of magnetic field in cross-sectional planes and estimate radial forces. In Figure 5S (a), the distribution of the value $\frac{d(B)^2}{dr}$ within the magnet volume is illustrated. The magnetic field vector lies in the plane of figure. Figures (b) and (c) show the distribution of magnetic field at cross sections within the warm bore of the magnet, specifically at vertical coordinates of 0 cm, where the field is uniform, and at -5 cm, where the lift force is at its maximum. Figures (d) and (e) illustrate the distribution of $\frac{d(B)^2}{dr}$ at these same cross sections.

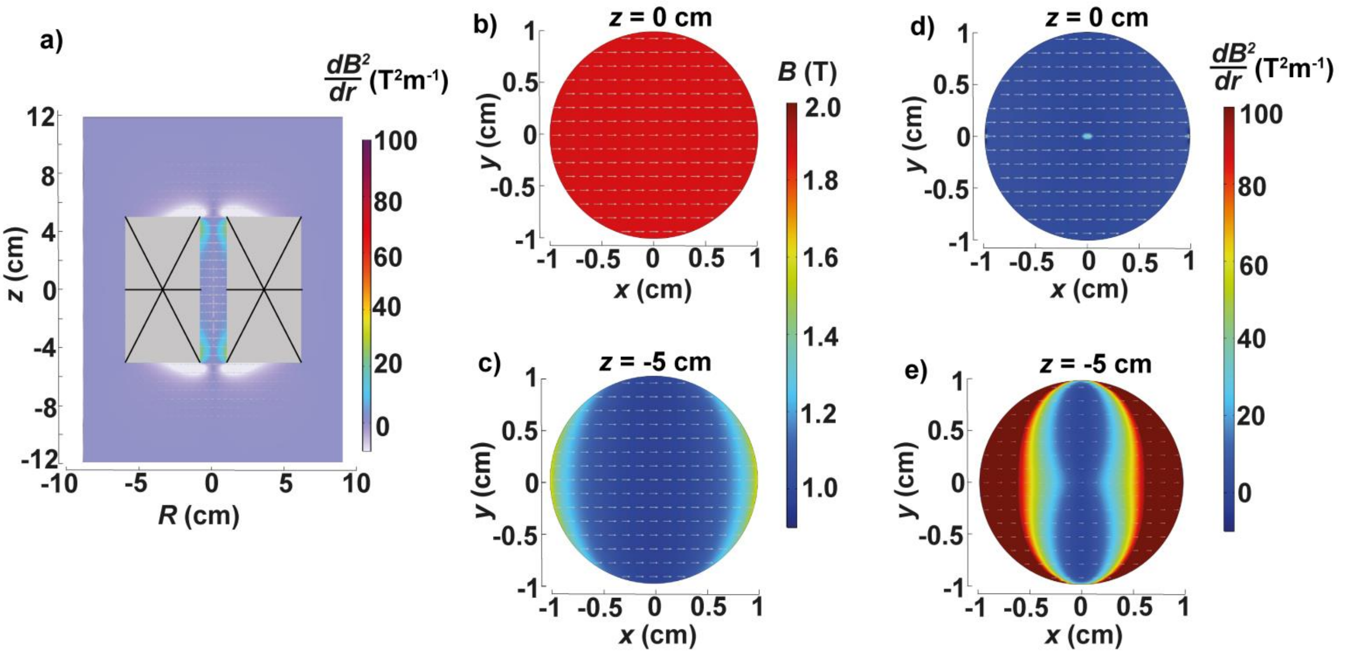

Figure 5S. The radial force characteristics of the Halbach magnet with outer diameter 120 mm. (a) The distribution of the value $\frac{d(B)^2}{dr}$ within the volume of the magnet. The magnetic field vector lies in the plane of the figure; (b) the distribution of magnetic field at cross sections within the warm bore of the magnet, specifically at vertical coordinates of 0 cm, where the field is uniform; (c) the distribution of magnetic field at -5 cm, where the magnetic lift force is at its maximum; (d) and (e) the distribution of $\frac{d(B)^2}{dr}$ at these same cross sections at 0 cm (d) and -5 cm (e).

The figure shows that this Halbach magnet configuration generates a radial force, approximately equal to the lifting force (~ 70 T^2m^{-1}) at a distance of about $r = 0.5$ cm from the magnet axis, particularly at the point with the maximum lifting force $z = 5$ cm. Consequently, during the crystal growth process within test tubes having a significant diameter (> 1 cm), the pressure force exerted on the vessel wall will be of a magnitude comparable to the force of gravity. Nevertheless, it is worth noting that significant wall pressure does not eliminate the potential for spatial differentiation of compounds throughout the growth process.

Thus, the magnitude of values B and $\frac{d(B)^2}{dz}$ of simulated magnets are much smaller than the magnitudes for the 300 MHz magnet. In addition, the results of the performed simulations show that the linear dimension of the zone with high value $\frac{d(B)^2}{dz}$ does not exceed 2 cm and 4 cm for magnets with outer diameter 12 cm and 32 cm, respectively.

For shielded superconducting magnets of NMR spectrometers Bruker NMR 200 MHz, Bruker 7400 MHz and 700 MHz, the field strength was measured only on the axes of the magnets. The values of the maximum magnetic field B, as well as the value $\frac{d(B)^2}{dz}$ on the magnet axis are given in Table 1S. The Table 1 also shows the values for simulated magnets with outer diameter 120 mm and 320 mm.

Table 1S. The magnitudes of the maximum magnetic field B and the value $\frac{d(B)^2}{dz}$ for superconducting magnets of NMR spectrometers Bruker 200 MHz, Bruker 300 MHz, Bruker III HD 400 MHz and Bruker 700 MHz and for permanent magnets considered in the text above the table.

Magnet type	Maximum magnetic field, T	Maximum value $\frac{d(B)^2}{dz}$, $T^2 \cdot m^{-1}$
Magnet of Bruker 200 MHz (UltraShield)	4.7	300 (20)
Magnet of Bruker 300 MHz (unshielded)	7.0	510 (20)

Magnet of Bruker 400 MHz (UltraShield)	9.4	1000 (60)
Magnet of Bruker 700 MHz (UltraShield)	16.4	2500 (150)
Vertically magnetized magnet 120 mm	0.42	17.6
Vertically magnetized magnet 320 mm	0.38	7.6
Halbach magnet 120 mm	1.62	71

The table illustrates that the value of $\frac{d(B)^2}{dz}$ is much bigger for superconducting magnets compared to permanent magnets. It is worth noting that the configuration of the permanent magnets considered in this work may not be optimal for attaining the highest value of $\frac{d(B)^2}{dz}$.

B3. The conditions for compensating gravitational force in the case of paramagnetic species vary with their magnetic susceptibility and the characteristic $\frac{d(\boldsymbol{B})^2}{dz}$ ***of the magnet***

It is possible to estimate the sufficient value of $\frac{d(B)^2}{dz}$ of the magnet so that various compounds can levitate in its magnetic field. From formula (2) for the force f, the required for levitation value $\frac{d(B)^2}{dz}$ can be expressed as following:

$$\frac{d(B)^2}{dz} = (\rho_b - \rho_s)\cdot\left(\frac{2g\mu_0}{\chi_b^V - \chi_s^V}\right) \quad (16S)$$

Where ρ_b is density of paramagnetic crystal, ρ_s is density of solution in which the crystal is placed, g is gravitational acceleration which in calculations is taken equal to 9.8 N / kg, χ_b^V is volume magnetic susceptibility of body immersed in solution, χ_s^V is volume magnetic susceptibility of solution. B is magnetic field, μ_0 is vacuum permeability which is equal to 1.25663706212 (19) × 10^{-6} N / m.

Figure 6S illustrates the relationship between the value of $\frac{d(B)^2}{dz}$ and the difference in magnetic susceptibility between the crystal and the solvent $(\chi_b^V - \chi_s^V)$ for crystals with varying differences in density between the crystal and the solvent $\Delta\rho = (\rho_b - \rho_s)$. The figure also displays the maximum values of $\frac{d(B)^2}{dz}$ for superconducting magnets of NMR spectrometers 300, 400, 500, and 700 MHz, as well as for permanent magnets with outer diameters of 120 mm and 320 mm. The calculations were performed for three different types of crystals: $CuSO_4 \cdot 5H_2O$, $CoSO_4 \cdot 7H_2O$, and $FeSO_4 \cdot 7H_2O$ in their saturated solutions at 25°C, as well as in distillated water.

The volume susceptibility of paramagnet is proportional to $\chi^V \sim \frac{N}{V}$, where N is number of spins and V is the volume of species. Dissolution does not change the number of spins in the system; therefore, we estimate the magnetic susceptibility of the solution using the following formula:

$$\chi_s^V = \chi_b^V \frac{V_b}{V_s} \quad (17S)$$

Where V_b is the volume of dissolved species, V_s is the volume of solution. This formula can be rewritten in terms of masses and densities:

$$\chi_b^V = \chi_b^V \frac{m_b\cdot\rho_s}{(m_b+m_l)\cdot\rho_b} = \chi_b^V \frac{\alpha\cdot\rho_s}{(\alpha+1)\cdot\rho_b} \quad (18S)$$

Where m_b is mass of species, m_l is mass of the solvent, ρ_s is density of the solution, ρ_b is the density of the body. The coefficient is entered in the formula: $\alpha = \frac{m_b}{m_l}$.

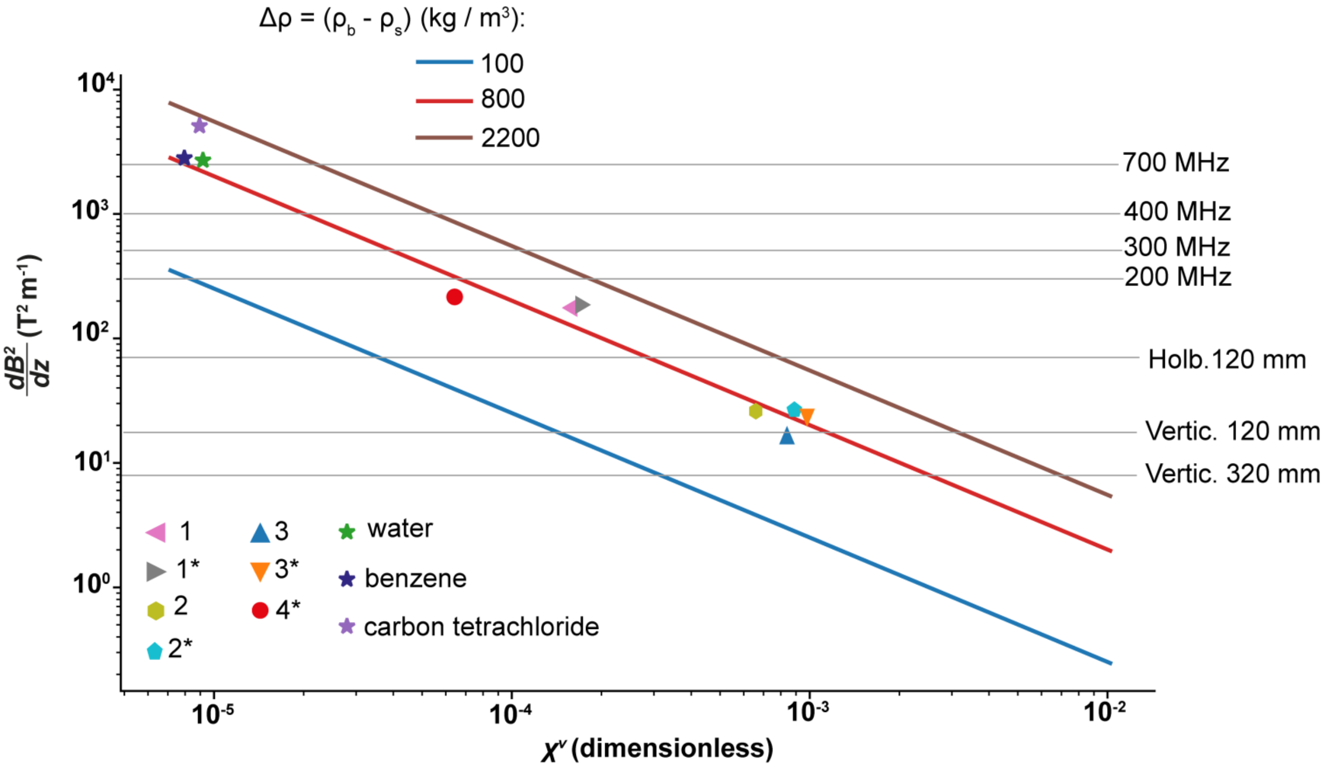

Figure 6S. Relationship between the required value $\frac{d(B)^2}{dz}$ for compensating the gravitational force and the difference between $\chi_b^V - \chi_s^V$ for species within the solutions with different $\Delta\rho = (\rho_b - \rho_s)$. Explanation of symbols on the chart: $CuSO_4 \cdot 5H_2O$ in a saturated water solution at room temperature (labeled 1), $CuSO_4 \cdot 5H_2O$ in distilled water at room temperature (labeled 1*), $CoSO_4 \cdot 7H_2O$ in saturated water solution at room temperature (labeled 2), $CoSO_4 \cdot 7H_2O$ in distilled water at room temperature (labeled 2*), $FeSO_4 \cdot 7H_2O$ in saturated water solution at room temperature (labeled 3), $FeSO_4 \cdot 7H_2O$ in distilled water at room temperature (labeled 3*), $L^{Me\text{-}CP}$ in hexane labeled (4*)[4]. The horizontal gray lines represent the maximum values of $\frac{d(B)^2}{dz}$ for various magnets, including those from NMR spectrometers 200, 300, 400, and 700 MHz, a Halbach magnet with an outer diameter of 120 mm (Holb. 120 mm), as well as vertically magnetized magnets with outer diameters of 120 mm and 320 mm (Vertic. 120 mm and Vertic. 320 mm respectively). The asterisks indicate the required value $\frac{d(B)^2}{dz}$ to compensate for compensating gravitational force for pure solvents: water, benzene, carbon tetrachloride.

The graph provides several significant conclusions. Firstly, superconducting magnets enable the attainment of gravitational force compensation for a broad range of paramagnetic compounds. In contrast, the permanent magnets considered in this work can only achieve compensation for high-spin compounds ($FeSO_4 \cdot 7H_2O$ and $CoSO_4 \cdot 7H_2O$ with spin S = 3/2). Secondly, solvent levitation can only be achieved using superconducting magnets with substantially higher maximum magnetic field values compared to the 300-MHz magnet discussed in detail in this work. Thirdly, the distinction between examining a crystal in a saturated solution versus distilled water is insignificant when estimating the essential parameters of the magnetic system. Thus, when calculating the position of the sample in the magnet for compensating gravitational force, the magnetic properties of solvent can be considered neglecting the contribution from dissolved paramagnetic species.

B4. The conditions for compensating gravitational force in the case of paramagnetic species vary with their molar mass and the characteristic $\frac{d(B)^2}{dz}$ of the magnet

It is advantageous to examine the correlation between the necessary parameters of the magnetic system not only in relation to the magnetic susceptibility of the compound but also in relation to the total spin of the compounds and their molar mass. To do this, it is necessary to rewrite the value χ in terms of spin and molar mass of species.

Molar magnetic susceptibility of some paramagnetic body can be written as:

$$\chi^m = \frac{N_A \cdot s(s+1) \cdot (g_f \cdot \beta)^2}{3kT} \qquad (19S)$$

Where N_A is Avogadro constant, μ_0 is vacuum permeability, g_f is g-factor, β is Bohr magneton, s is spin of the species, k is Boltzmann constant, T is temperature. The molar magnetic susceptibility at constant temperature is determined only by the spin of the molecules of a given compound and the g-factor, which has the value of about 2 for a large list of compounds. Molar magnetic susceptibility and volume magnetic susceptibility are linked by the following ratio:

$$\chi^V = \chi^m \frac{\rho}{M} \qquad (20S)$$

Where M is molar mass and ρ is density. Thus, it is possible to calculate the value $\frac{d(B)^2}{dz}$, which is necessary for the levitation of paramagnetic bodies, depending on their molar mass This estimation incorporates not only the molar mass but also the ratio of the densities of the solution and the submerged body $\frac{\rho_s}{\rho_b}$. Figure 7S shows the dependence of $\frac{d(B)^2}{dz}$ on molar mass necessary for levitation of compounds and their solutions with different densities of species ρ_b, solutions ρ_s. Figure 7S (a) represents the value $\frac{d(B)^2}{dz}$ required for levitation of the system with a spin of 1/2, while figure 7s (b) corresponds to the levitation of the system with a spin of 3/2. The positions of the crystals $CuSO_4 \cdot 5H_2O$, $L^{Me\text{-}CP}$ heterospin complex[4] with spin 1/2 as well as $CoSO_4 \cdot 7H_2O$ and $FeSO_4 \cdot 7H_2O$ with spin 3/2, are marked on the graphs (a) and (b) respectively. The magnets used in the experiment are marked with signatures on the $\frac{d(B)^2}{dz}$ - value scale, which correspond to the magnetic field strength required to counterbalance the force of gravity for compounds possessing a spin of 1/2 (a) and 3/2 (b).

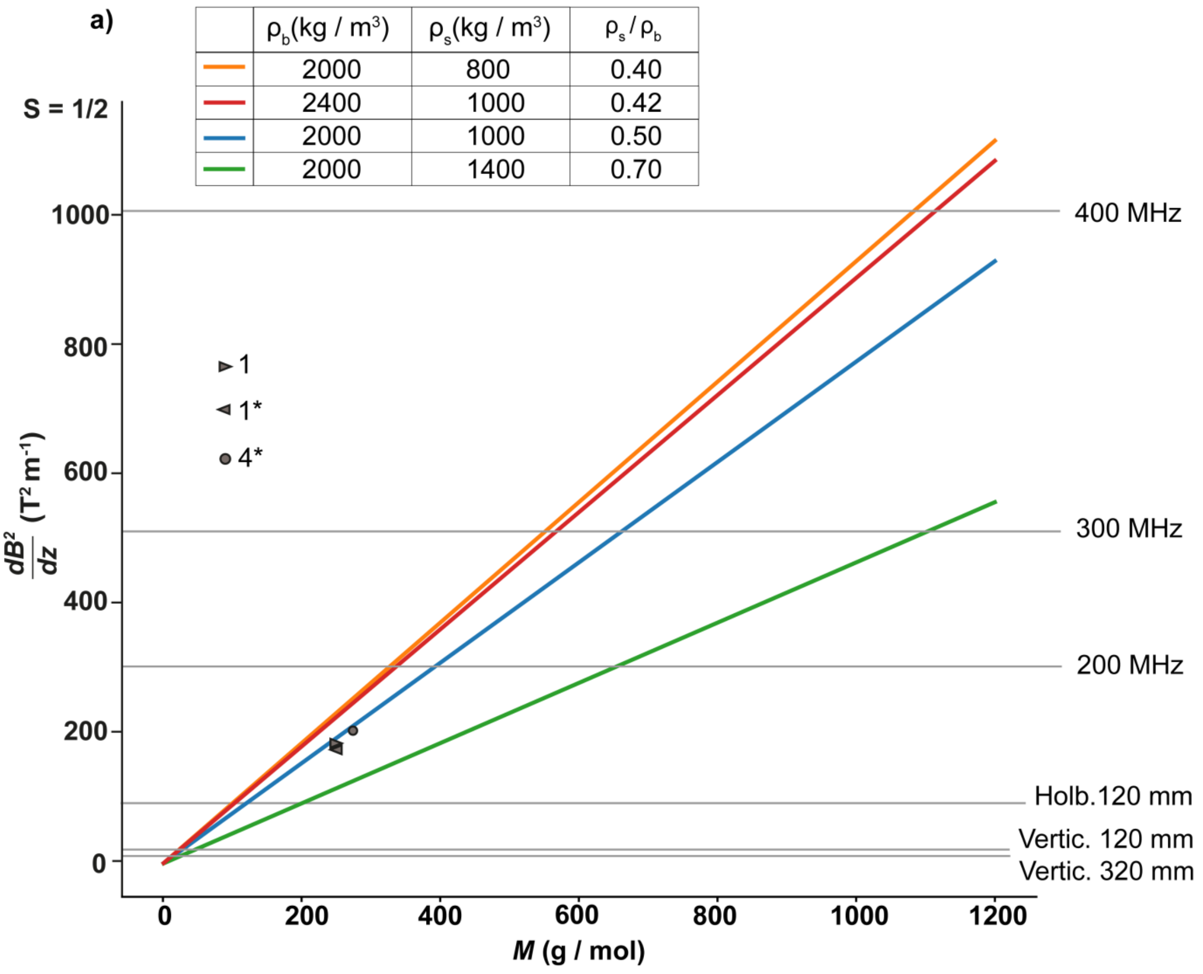

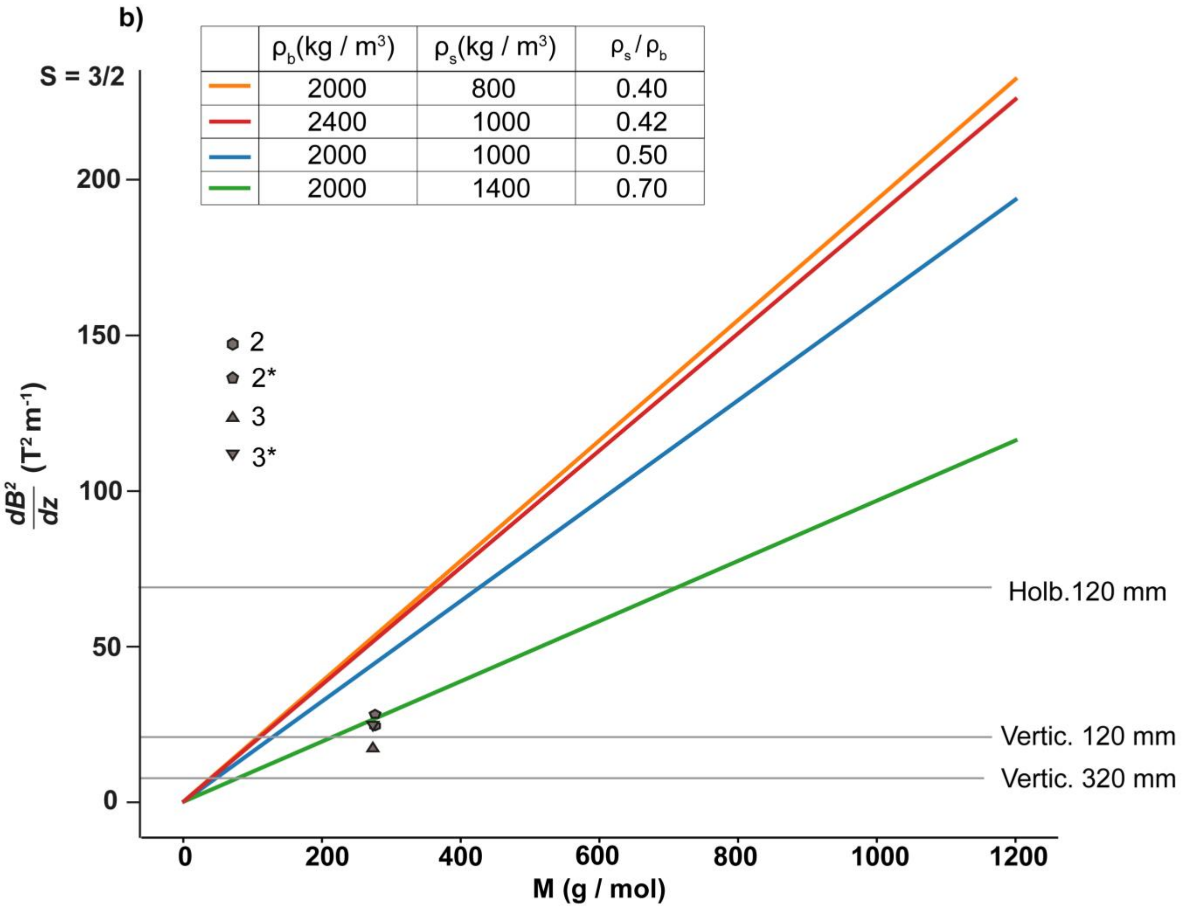

Figure 7S. Relationship between the required value $\frac{dB^2}{dz}$ for compensating the gravitational force and molar mass of species in the solutions with different densities of body (ρ_b) and density of solution (ρ_s). The density of a solution (ρ_s) at 1000 kg / m^3 matches that of water, while a density of 800 kg / m^3 is close to typical for organic solvents. The value 1400 kg/m^3 corresponds to saturated aqueous salt solutions. Figure (a) refers to values associated with compounds having a spin of 1/2, figure (b) corresponds to those with a spin of 3/2. Explanation of symbols on the chart: $CuSO_4 \cdot 5H_2O$ in a saturated water solution at room temperature (labeled 1), $CuSO_4 \cdot 5H_2O$ in distilled water at room temperature (labeled 1*), $CoSO_4 \cdot 7H_2O$ in saturated water solution at room temperature (labeled 2), $CoSO_4 \cdot 7H_2O$ in distilled water at room temperature (labeled 2*), $FeSO_4 \cdot 7H_2O$ in saturated water solution at room temperature (labeled 3), $FeSO_4 \cdot 7H_2O$ in distilled water at room temperature (labeled 3*), $L^{Me\text{-}CP}$ in hexane labeled (4*)[4]. The horizontal gray lines represent the maximum values of $\frac{d(B)^2}{dz}$ for various magnets, including those from NMR spectrometers 200, 300, 400, and 700 MHz, a Halbach magnet with an outer diameter of 120 mm (Holb. 120 mm), as well as vertically magnetized magnets with outer diameters of 120 mm and 320 mm (Vertic. 120 mm and Vertic. 320 mm respectively).

C. The design of the insert for crystallization in the magnet of the 300 MHz NMR spectrometer

Figure 8S shows the setup developed for experiments under controlled temperature conditions in the magnet of the 300 MHz NMR spectrometer. The setup includes a sample (1) placed in a thermostat in the magnet (5) and is temperature-stabilized using coolant (2) which is cooled by a Peltier cooler (6). The coolant is pumped using a pump (7). Two sensors (3) are used for temperature control: one is placed in the thermostat in the sample area and the other one is attached to cold side of the Peltier cooler**.** The Peltier cooler is supplied with power from a power source (8), and this power source is regulated and controlled by a thermal controller (9).

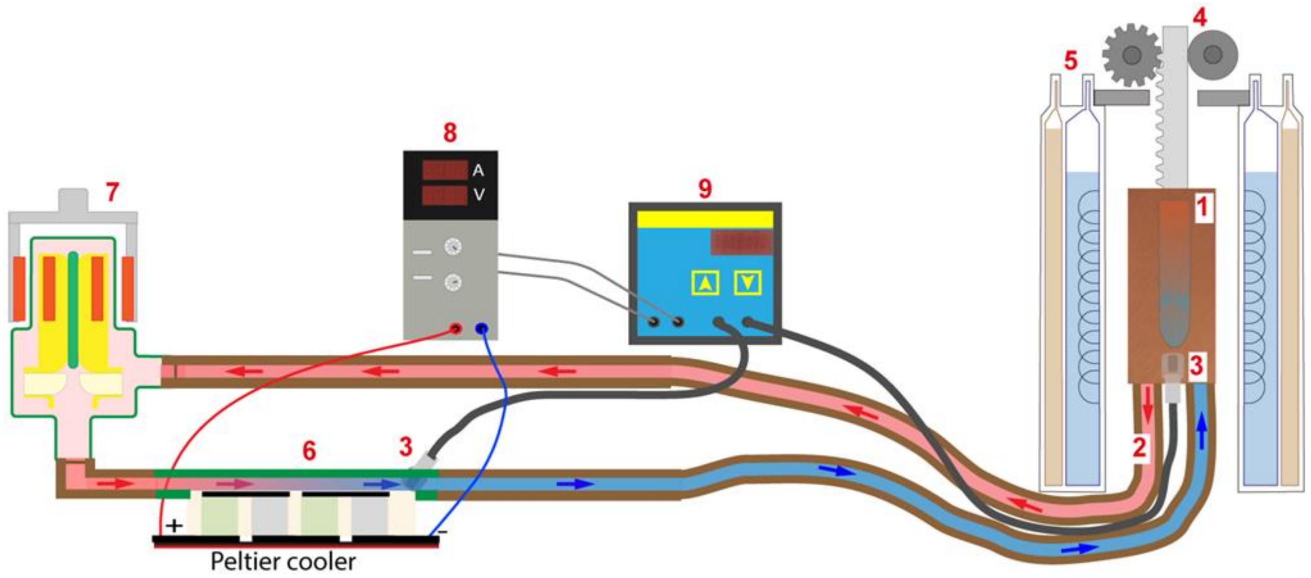

Figure 8S. The setup for crystal growth in the warm bore of superconducting magnet of NMR spectrometer 300 MHz. (1) Thermostat with a sample; (2) Heat carrier flow in the thermostat; (3) Temperature sensors; (4) Positioning system controlled by step engine; (5) NMR magnet.(6) Peltier cooler; (7) Pump; (8) Power source; (9) Temperature controller.

Figure 9S shows the sample positioning system: scheme of the system (a) and photos of the system from the top (b) and side (c) views. The positioning was achieved by using a step motor (1) which was connected to a rack (2) via a system of pinions and bearings. The rack was then attached to the thermostat containing the sample.

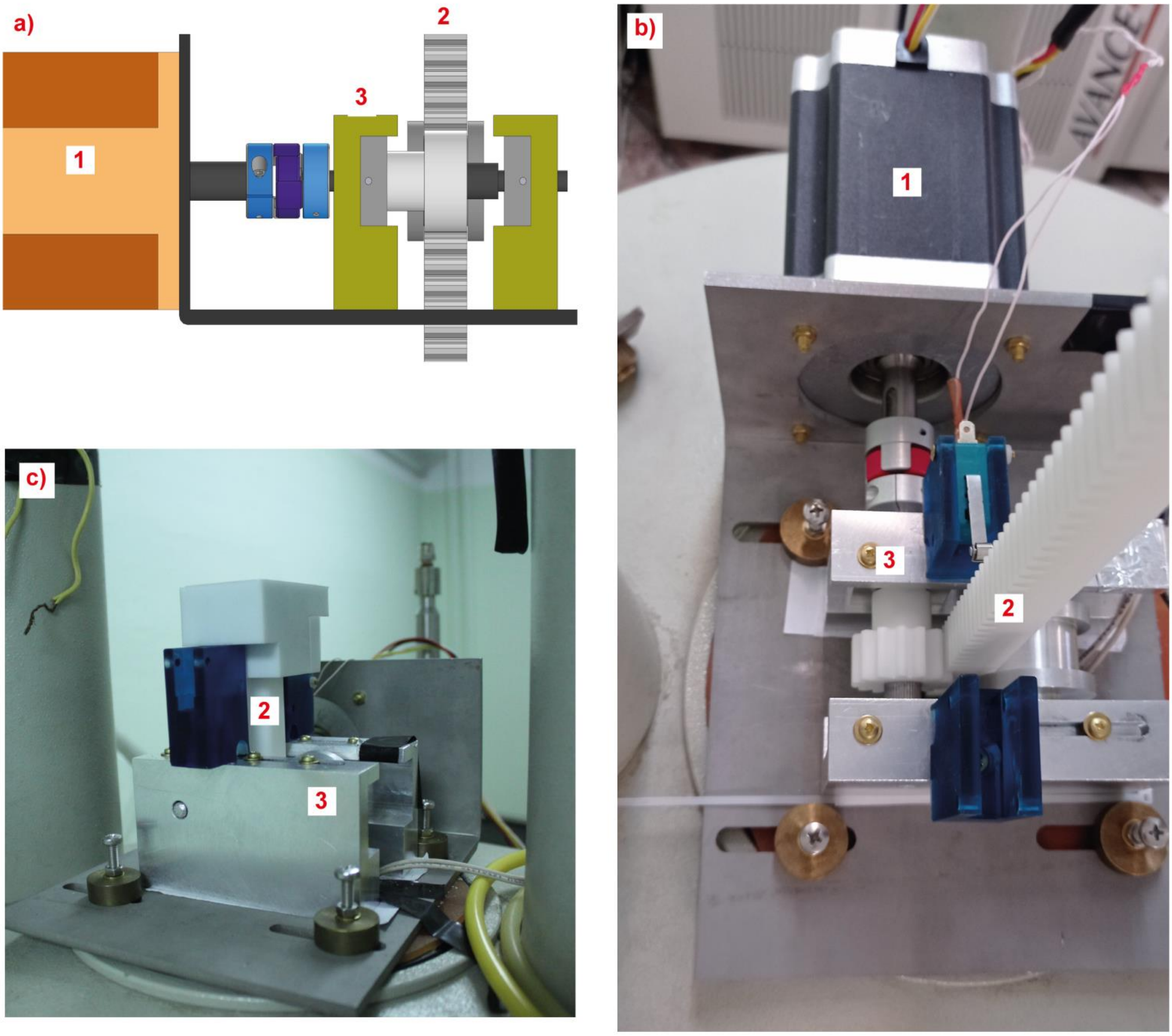

Figure 9S. The sample positioning system: scheme of the system (a) and photos of the system photos of the system from the top (b) and side (c) views. (1) Step motor; (2) Rack which is attached to the thermostat; (3) Bearings and pinions system.

Figure 10S shows the thermostatic setup block: without (a) and with (b) the external heat-insulating shell. The sample (1) was placed in the block (2) to which the coolant (3) was supplied. The outer shell (4) is put on the block, which was attached to the rock (5).

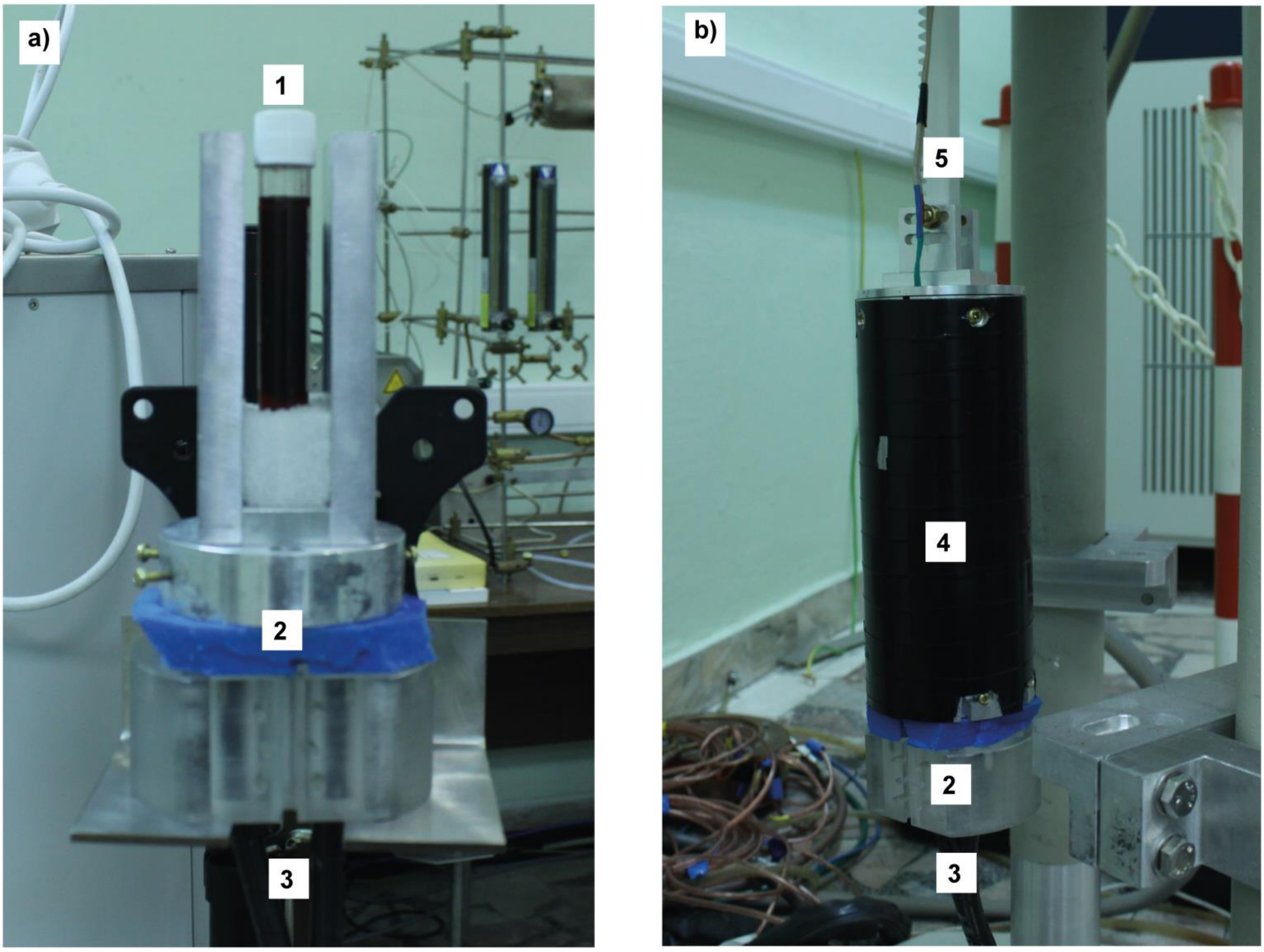

Figure 10S. Thermostatic setup block: without (a) and with (b) external heat-insulating shell. (1) sample; (2) block for sample mounting; (3) coolant; (4) heat-insulating shell; (5) rack.

D. Characterization of $CoSO_4 \cdot 7H_2O$ crystals

Figure 11S shows $CoSO_4 \cdot 7H_2O$ crystals grown in the absence of a magnetic field (a) and in its gradient (b). The crystals, which were grown in the magnetic field gradient, clogged the tube with the solution, and therefore a part of the solution was not drained. In the case of the absence of magnetic field, it was impossible to extract the crystals without crushing them. To make the extraction of the crystals from the tube easier, crystals were also grown in the absence of a magnetic field in a horizontal orientation of the test tube. Figure 12S (a) shows the typical crystals, which were obtained in the absence of magnetic field in the horizontal orientation of the tube. Figure 12S (b, c) shows obtained in the magnetic field gradient crystals.

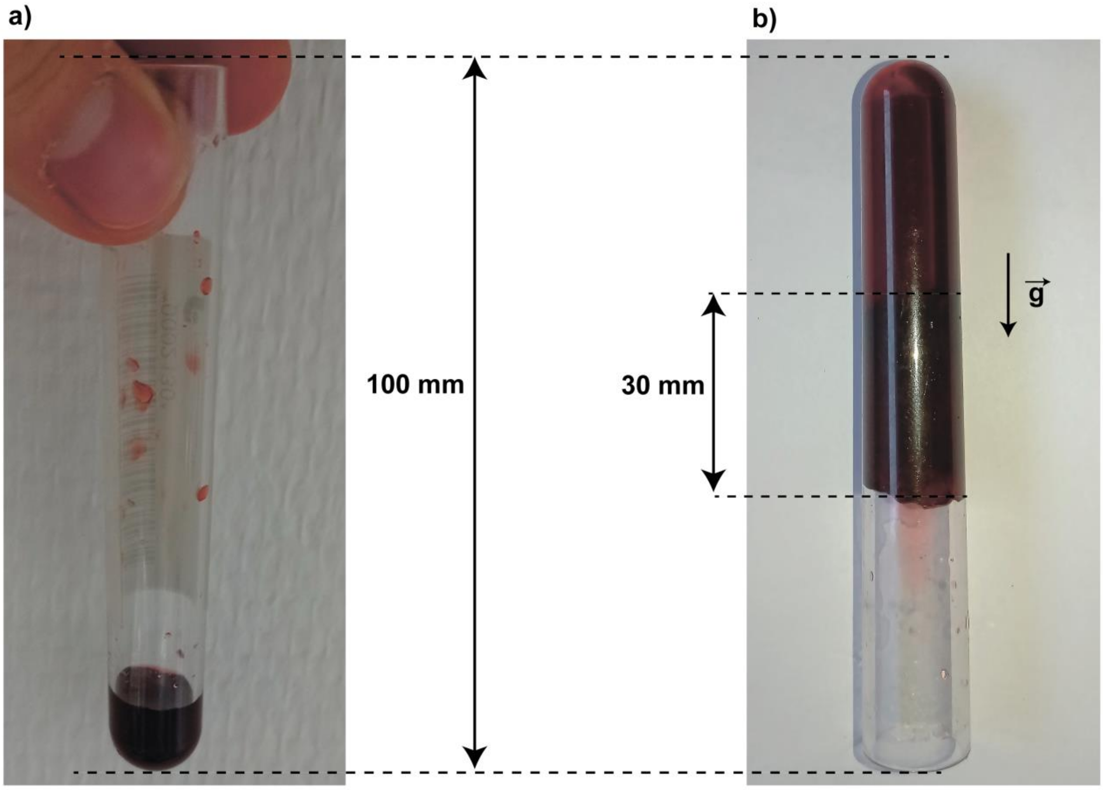

Figure 11S. Tubes with $CoSO_4 \cdot 7H_2O$ crystals. (a) Crystals were obtained in the absence of magnetic field; (b) crystals were obtained in the magnetic field gradient.

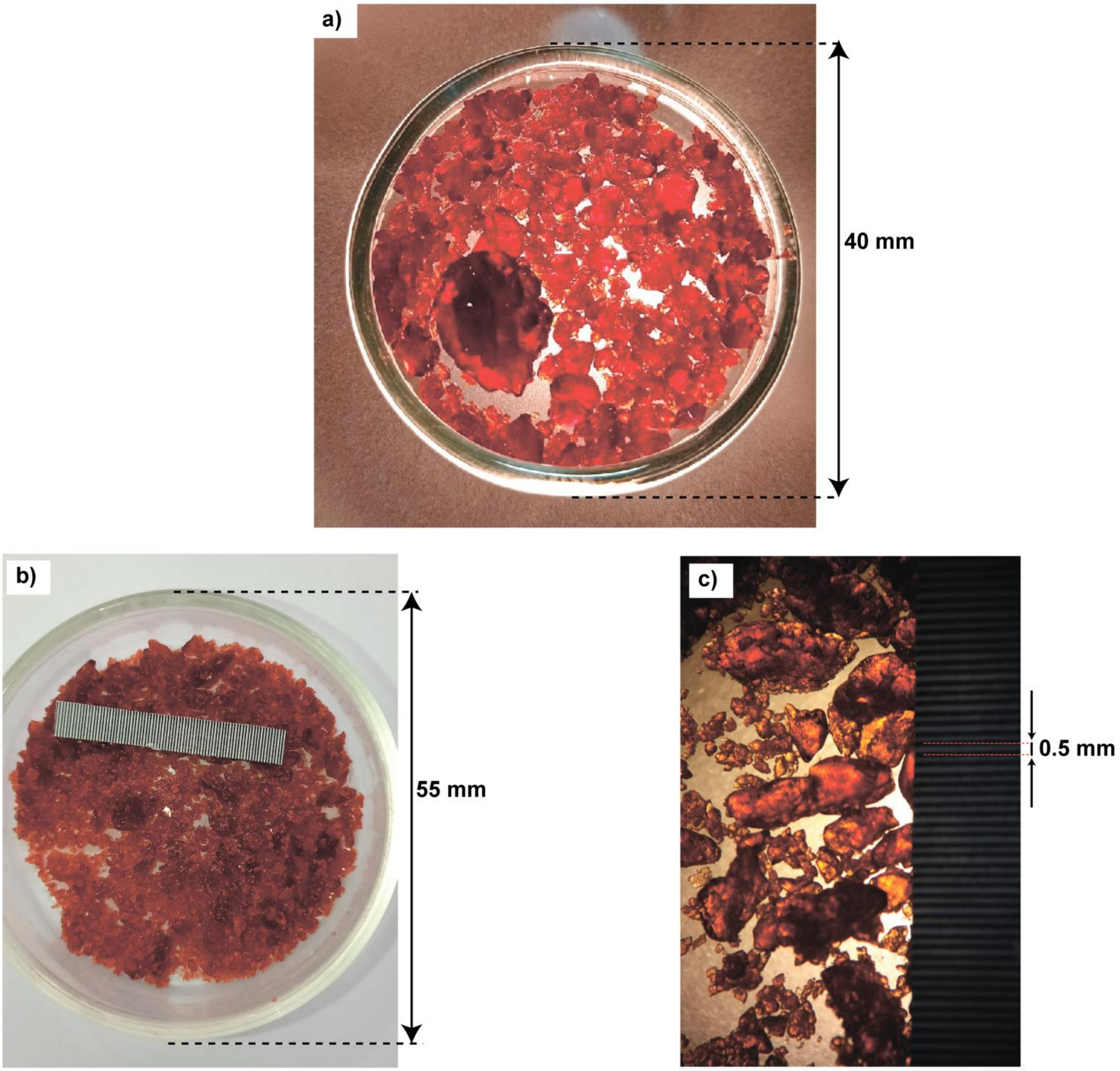

Figure 12S. Different sets of $CoSO_4 \cdot 7H_2O$ crystals. (a) shows crystals that were grown without the magnetic field in the horizontal orientation of the tube; (b) shows crystals that were grown in the presence of a magnetic field gradient; (c) shows a close-up image of crystals grown in the presence of a magnetic field under a microscope.

Photos of all grown crystals are shown in figure 13S. Photos (a-d) show crystals grown in a gradient magnetic field. The positions of all tubes were the same except for tube (d), which was located 1 cm higher than the others. Photos (e-f) indicate crystals grown in the absence of a magnetic field.

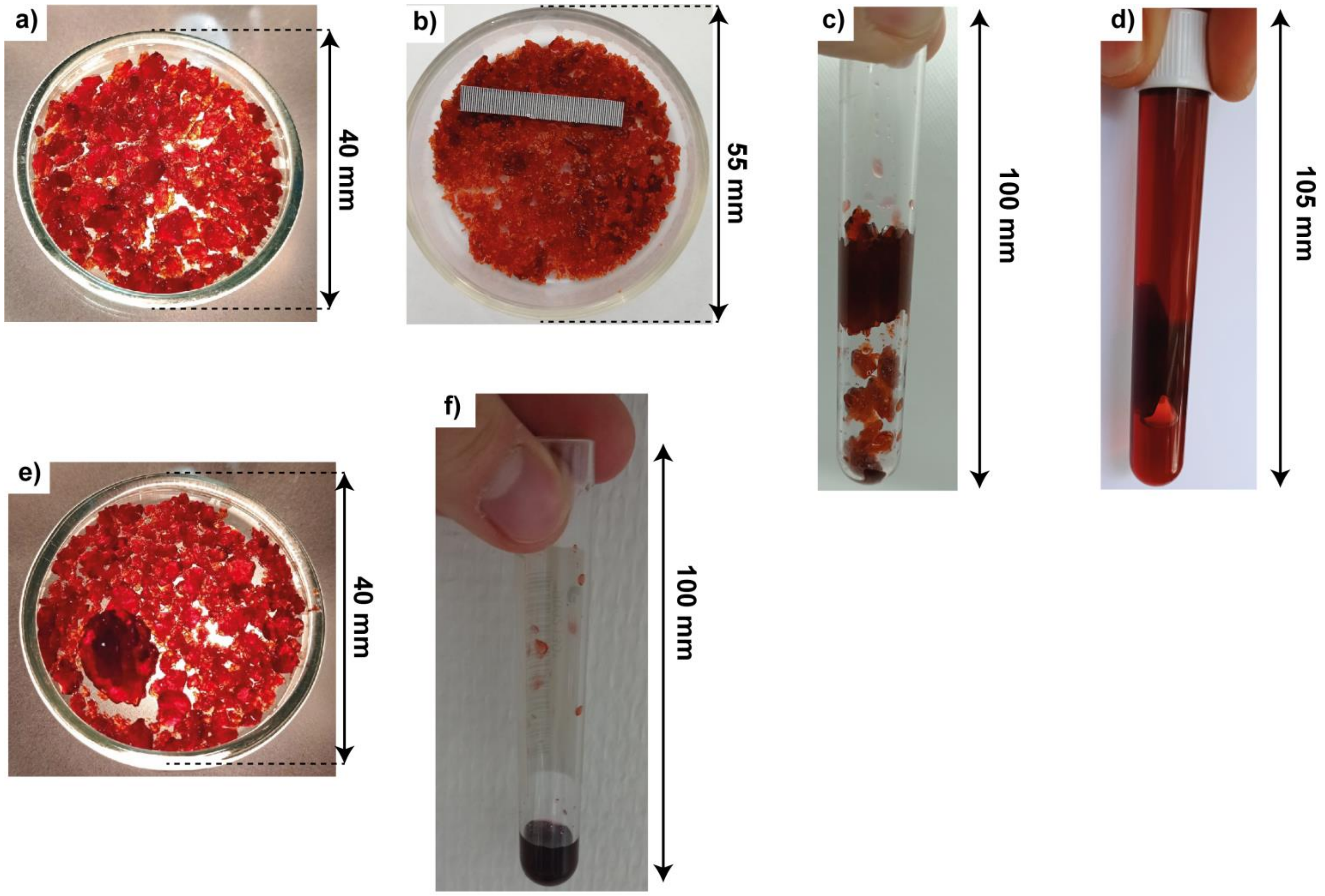

Figure 13S. $CoSO_4 \cdot 7H_2O$ crystals grown in the present magnetic field (a-d) and its absence (e-f).

In total, 4 experiments were carried out on growing crystals in the magnetic field gradient and 2 experiments on growing in its absence. Reliable differences between the sizes of crystals and its amount obtained in the magnetic field and in its absence were not revealed.

E. Detailed characterization of crystals grown from a solution of a mixture of copper and cobalt sulfates

Using a solution of a mixture of copper and cobalt sulfates, experiments were conducted involving a magnetic field gradient as well as the absence of a field, with 3 trials performed for each condition. In all instances, a CoSO4 · $7H_2O$ crystal served as the seed for the experiments. The growth outcomes for all experiments are visually presented in Figure 14S. Photos (a-c) show crystals grown in the absence of magnetic field and (d-f) in the gradient of magnetic field.

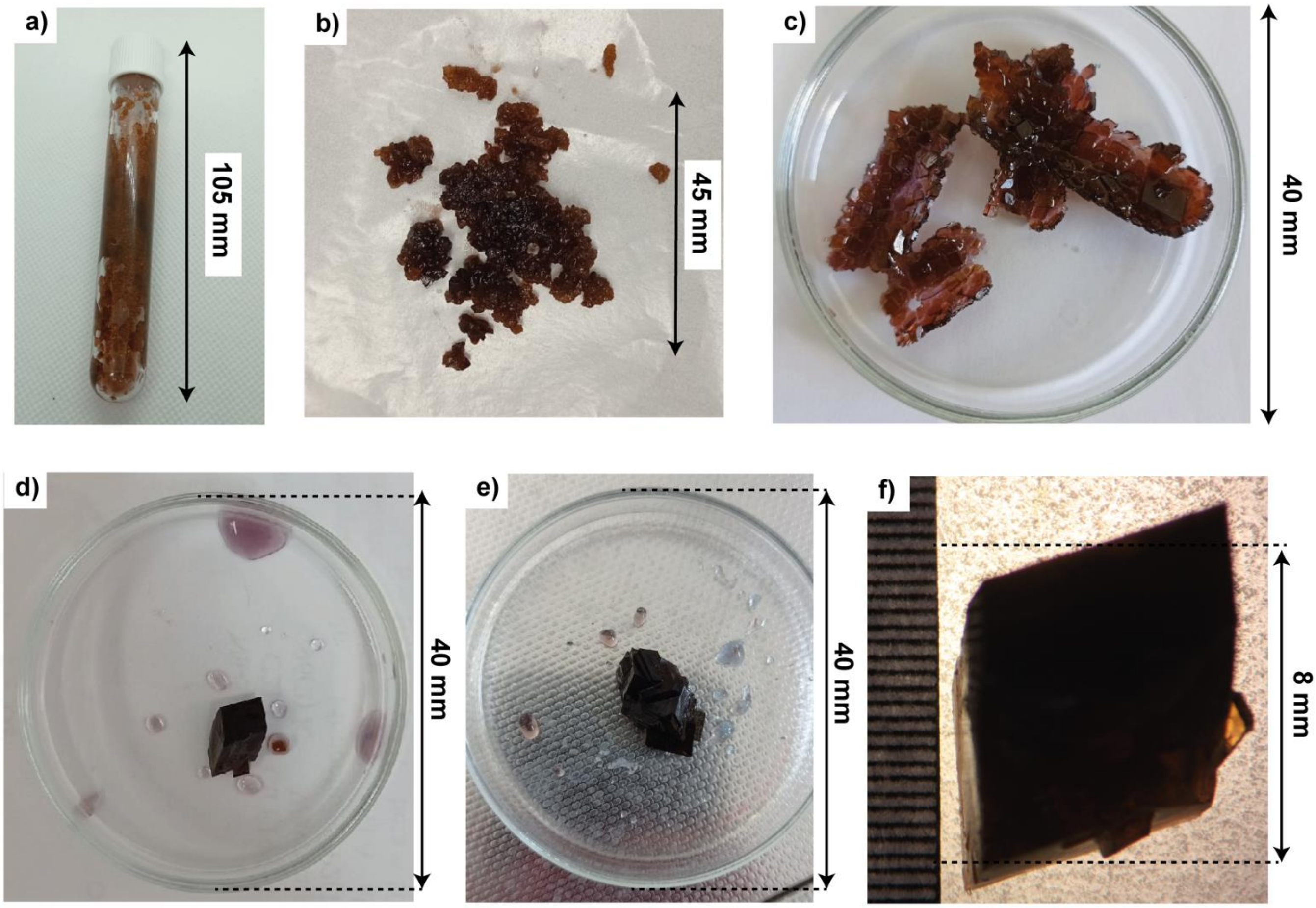

Figure 14S. Photos of crystals grown in a solution of a mixture of copper and cobalt sulfates in the absence of magnetic field (a-c) and in the gradient of magnetic field (d-f).

Figure 15S illustrates the recorded spectra on the UV-visible spectrophotometer. The spectra of cobalt sulfate and copper sulfate solutions are represented by the red and blue lines, respectively. The cyan and magnet lines correspond to the spectra of the mixed solution of copper and cobalt sulfates, normalized with respect to the cobalt sulfate solution line.

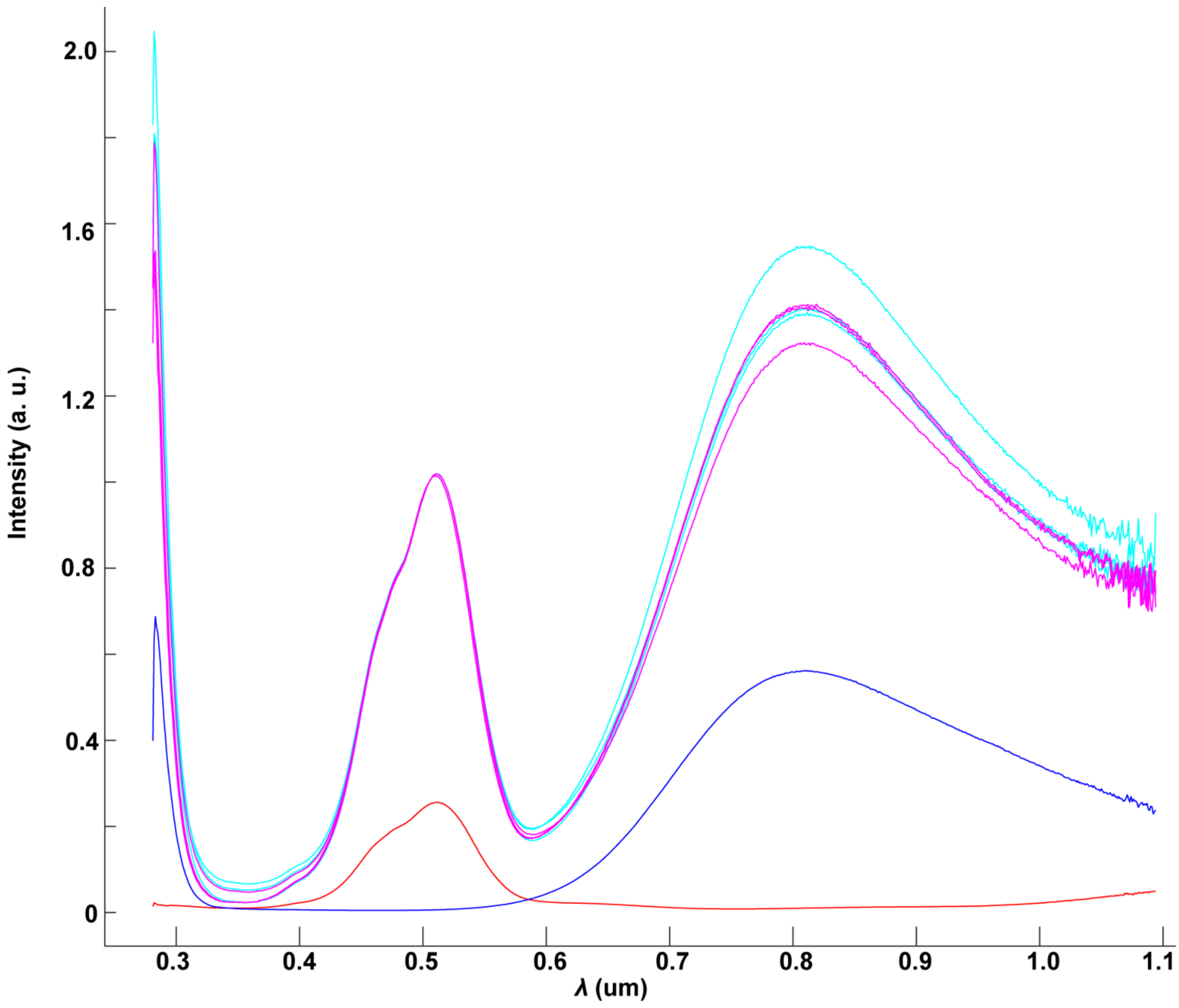

Figure 15S. The spectrophotometry data of dissolved crystalline samples grown from a solution of a mixture of copper and cobalt sulfates under magnetic field (—) and without magnetic field (—). The lines (—) and (—) represent the spectra of $CuSO_4 \cdot 5H_2O$ and $CoSO_4 \cdot 7H_2O$ water solutions respectively.

The spectrophotometry data does not allow for definitive conclusions to be drawn. The presence of a higher concentration of copper sulfate in one of the solutions may suggest the potential precipitation of a small quantity of $CuSO_4 \cdot 7H_2O$ crystals as a distinct phase.

F. The description of video materials

The positioning system was equipped with a step motor that allowed for oscillatory movements. As part of trial experiments, the effect of these rocking motions on the growth processes was investigated. The hypothesis was that such movements would prevent the crystals from sticking together and also from sticking to the wall of the tube. However, it was found that the rocking motion did not provide clear benefits, and therefore, the idea was abandoned and not included in the experiments described earlier. Nevertheless, some of the videos below were recorded while the rocking motion was applied.

Videos in good quality can be found on the following sites:

https://github.com/ArkadySamsonenkoWork/Article_Crystals_2023
https://www.youtube.com/watch?v=bxz_YvjEKvM&list=PLYtBZzLlCz0g5Y5xHRpPPMai9-JLeMc6v

1. The video shows the growth process of copper sulfate crystals on the inner wall of a test tube. The crystal growth occurred within the field of view of the pre-positioned video probes cameras.

2. The video shows the motion of copper sulfate immersed in water. The test tube containing the crystal is positioned within a strong magnetic field gradient, resulting in the achievement of gravitational compensation at the level of the video probes. Throughout the video, the sample oscillates within the magnet.

3. The video shows the process of positioning the test tube inside the magnet. Initially, a crystal of copper sulfate was lowered into a test tube with water.

G. Analysis of the influence of various effects on crystal growth in the magnetic field

G1. Estimation of magnetic dipole force influence

Consider two crystals of the size much smaller than the distance between them. In the magnetic field, paramagnetic crystals have magnetization, which leads to the interaction between them with the energy:

$$E = \frac{\boldsymbol{m_1} \boldsymbol{m_2}}{R^3} - 3\frac{(\boldsymbol{m_1 R})(\boldsymbol{m_2 R})}{R^5} \qquad (21S)$$

Where $\boldsymbol{m_1}$ and $\boldsymbol{m_2}$ are magnetic moments vectors of these crystals. $\boldsymbol{R}$ is radius-vector connecting both crystals. We will assume that the crystals are close enough to each other (nevertheless, the distance is greater than their sizes) so that the magnetic field in which the crystals are located is approximately the same. In such approximations, we can assume that:

$$\boldsymbol{m_1} = \frac{\chi^V V_1}{\mu_0}\boldsymbol{B} \qquad (22S)$$

$$\boldsymbol{m_2} = \frac{\chi^V V_2}{\mu_0}\boldsymbol{B} \qquad (23S)$$

Where χ^V is volume magnetic susceptibility, μ_0 is vacuum permeability, V is volume of a crystal. Then:

$$E = \left(\frac{\chi^V}{\mu_0}\boldsymbol{B}\right)^2 \frac{V_1 V_2}{R^3} - 3\left(\frac{\chi^V}{\mu_0}\boldsymbol{B}\right)^2 \frac{V_1 V_2}{R^3} \cdot cos(\alpha) = \left(\frac{\chi^V}{\mu_0}\boldsymbol{B}\right)^2 \frac{V_1 V_2}{R^3}\left(1 - 3cos^2(\alpha)\right) \qquad (24S)$$

Where α is angle between radius-vector $\boldsymbol{R}$ and magnetic field $\boldsymbol{B}$. Now it is possible to find force acting on the crystal with magnetic moment $\boldsymbol{m}_2$ from the crystals with the magnetic moment $\boldsymbol{m}_1$. $\boldsymbol{R}$ in this case will direct from $\boldsymbol{m}_1$ to $\boldsymbol{m}_2$.

$$\boldsymbol{F} = \left(\frac{\chi^v}{\mu_0}\boldsymbol{B}\right)^2 \frac{V_1 V_2}{R^4}(1 - 3cos^2(\alpha)) \cdot \frac{\boldsymbol{R}}{R} \qquad (25S)$$

Let us estimate the value of $\left(\frac{\chi^v}{\mu_0}\boldsymbol{B}\right)^2 \frac{V_1 V_2}{R^4}$. We assume that the crystals are of the same size:

$$V_1 = V_2 = V \qquad (26S)$$

As estimation, we take the values of B, χ^V, ρ for $CoSO_4 \cdot 7H_2O$ in the form:

$\rho = 1.95\frac{g}{cm^3}$,

$\chi^V \approx 871 \cdot 10^{-6}$,

At the point of $CoSO_4 \cdot 7H_2O$ growth: B $\approx$ 7 T

The distance between the crystals R is taken equal to 1 cm, which corresponds to the diameter of the test tube used in the experiment and the characteristic scale of the change of value B in the magnet of spectrometer 300 MHz.

Comparison of the speed of thermal motion $\sqrt{\frac{3kT}{m}}$ of crystals with the speed of drift caused by the magnetic dipole interaction shows that the last speed begins to dominate at a volume of crystals more then $V = (0.04\,mm)^3$. The speed of drift was estimated using formula $v = \left(\frac{\chi^V}{\mu_0}\boldsymbol{B}\right)^2 \frac{V^2}{R^4} \cdot \mu$, where μ is mobility, estimated via the Stoke's law, v is speed of drift. When two crystals are randomly positioned relative to each other, the factor $1 - 3cos^2(\alpha)$ is more likely to be negative, which results in attraction between the crystals.

Thus, under conditions of compensation of the gravitational force in the magnetic field gradient, the drift caused by the magnetic dipole interaction can also affect the processes of crystal growth in the presence of several crystallization centers. More likely attractive magnetic dipole interactions may facilitate the aggregation of these crystallization centers and the formation of one crystal.

G2. Estimation of test tube off-axis displacement effect on possible aggregation of crystal centers

Let us consider a crystal in solution, located on the wall of the test tube with a sample. The figure shows a horizontal section of this test tube. The center of the test tube is shifted relative to the axis of a magnet warm bore symmetry by the

distance Δr. The radius of the tube is R. The forces acting on the crystal: force from the magnetic field F_m, the reaction force N, and the force of friction F_{fr}.

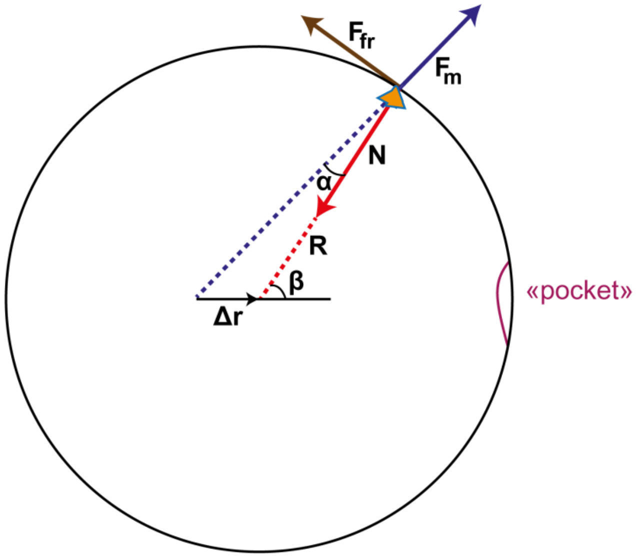

Figure 16S. Paramagnetic crystal on the wall of the test tube. F_{fr} is friction force, N is reaction force, F_m is force from the magnetic field, Δr is shift of the test tube respectively to the axis of magnet warm bore symmetry, R is the test tube radius.

From the equilibrium condition of the crystal:

$$\frac{(F_m)_\tau}{N} = tg(\alpha) \qquad (27S)$$

Where $(F_m)_\tau$ is tangential component of the magnetic field force F_m. On the other side:

$$cos(\alpha) = \frac{\frac{\Delta r}{R}\cdot\cos(\beta)+1}{\sqrt{(\frac{\Delta r}{R})^2+2\frac{\Delta r}{R}\cdot\cos(\beta)+1}} \qquad (28S)$$

This expression reaches its minimum (maximum of the value $tg(\alpha)$) at $\cos(\beta) = 0$. Then the maximum value $tg(\alpha)$:

$$\big(tg(\alpha)\big)max = \frac{\Delta r}{R} \qquad (29S)$$

If the radius of the tube is 7mm, and Δr is at least 1mm, then the maximum value of $\big(tg(\alpha)\big) = 0.14$. This value is greater than the friction coefficient between many materials. Additionally, since the crystal is in an aqueous solution, the friction between the test tube and crystal is reduced. Therefore, the shift of the test tube relative to the axis of magnet symmetry could indeed be the cause of the formation of a "pocket" in an area with an angle β ~ 0 (fig. 16S). This pocket serves as a region where crystallization centers are attracted and form in such way one crystal.

It is worth noting that in the conducted experiments, a predominant formation of crystals on one side of the vessel wall was not observed in the presence of a magnetic field. This suggests that the test tube off-axis displacement did not appear to be the primary factor influencing the formation of one crystal in the magnetic field.

G3. Estimation of convection suppression effect

Following the article[5] let's consider the effect of a magnetic field gradient on natural convection in a solution. The mechanism of convection during crystal growth is similar to that in a liquid with a temperature gradient. In the depletion zone near the crystal, the concentration of the dissolved paramagnetic species is lower than in bulk, as a result, the density of the solution is also lower. It leads to the non-zero buoyancy force acting to this part of the solution. Effective force can be written in form[5]:

$$F_{eff} = \frac{\Delta\chi_s^V}{2\mu_0}\frac{d(B)^2}{dz} - \Delta\rho_s g \qquad (30S)$$

Where $\Delta\chi_s^v$ and $\Delta\rho_s$ are the differences between volume magnetic susceptibility and density of bulk and crystal interface. Under low difference between concentration at crystal interface and bulk these values can be written in form of:

$$\Delta\chi_s^V = \chi_b^V(1+\Delta\alpha) \qquad (31S)$$

$$\Delta\rho_s = \rho_b(1+\Delta\alpha) \qquad (32S)$$

In this case the effective acceleration of the solution is given by:

$$g_{eff} = -\frac{F_{eff}}{\Delta\rho_s} = g(1-\frac{\chi_b^V}{2\rho_b\mu_0}\frac{dB^2}{dz}) \qquad (33S)$$

In the zone of crystal growth:

$$\frac{\chi_b^V}{2\mu_0}\frac{dB^2}{dz} = \rho_b - \rho_s \qquad (34S)$$

So, close to crystal

$$g_{eff} = g\frac{\rho_s}{\rho_b} \qquad (35S)$$

For the low concentrated aqueous solutions of $CoSO_4 \cdot 7H_2O$ and $CuSO_4 \cdot 5H_2O$ these values are $0.51g$ and $0.43g$ respectively, and for saturated solutions at 25°C they are $0.69g$ and $0.52g$.

As was shown in the article[6], these values of g_{eff} significantly greater than the value of total compensation of convection. Nevertheless, decrease of g_{eff} leads to reduction of depletion zone[5] and reduction of convection. Free convection, being one of the mechanisms of mass exchange, has a significant effect on the growth rate of crystals[7]. Therefore, partial suppression of convection in the magnetic field gradient can lead to slower crystal growth.

H. Crystallographic characteristics and crystallization conditions of $Cu_xCo_{1-x}SO_4 \cdot 7H_2O$

Table 2S. The Crystallographic characteristics and conditions of crystallization of $Cu_xCo_{1-x}SO_4$ $7H_2O$

Compound	NA-Cu-Co22	NA-cu-co23	“literature”
x_{Cu}	~0.4	~0.4	0.46[8]
B, T	**~7**	Without magnetic field	Without magnetic field
Space group, *Z*		$P\,2_1/c$, 4	
a,	14.0893(6)	14.1049(3)	14.0875(12)
b,	6.5120(3)	6.51740(10)	6.5124(5)
c, Å	10.8251(5)	10.8092(2)	10.8397(9)
β, °	105.548(3)	105.6270(10)	105.509(1)
V, $Å^3$	956.85(8)	956.93(3)	958.26(14)
D_c, g cm^{-3}	1.960	1.961	1.950
θ_{max}, deg.	29.542	29.600	28.78
I_{hkl} (meas/uniq)	10159 / 2653	9061 / 2659	11 348 / 2367
R_{int}	0.0187	0.0239	0.0352
$I_{hkl}(I>2\sigma_I)$ / Ns	2290 / 164	2000 / 164	–
GooF	1.061	1.090	1.038
R_1 / wR_2 ($I>2\sigma_I$)	0.0231 / 0.0581	0.0315 / 0.0738	0.0232 / 0.0608
R_1 / wR_2 (*all data*)	0.0283 / 0.0608	0.0474 / 0.0814	0.0261 / 0.0623
code	2339711 (CSD)	2339712 (CSD)	156698 (ICSD)

I. References

(1) Koustav Banerjee. Inequalities for the Modified Bessel Function of First Kind of Non-Negative Order. https://doi.org/10.13140/RG.2.2.31023.61604

(2) Ravaud, R.; Lemarquand, G. Discussion about the Magnetic Field Produced by Cylindrical Halbach Structures. *Prog Electromagn Res B* **2009**, *13*, 275–308. https://doi.org/10.2528/PIERB09012004

(3) Doğan, N.; Topkaya, R.; Subaşi, H.; Yerli, Y.; Rameev, B. Development of Halbach Magnet for Portable NMR Device. *J Phys Conf Ser* **2009**, *153*, 012047. https://doi.org/10.1088/1742-6596/153/1/012047

(4) Tolstikov, S. E.; Artiukhova, N. A.; Romanenko, G. V.; Bogomyakov, A. S.; Zueva, E. M.; Barskaya, I. Y.; Fedin, M. V.; Maryunina, K. Y.; Tretyakov, E. V.; Sagdeev, R. Z.; Ovcharenko, V. I. Heterospin Complex Showing Spin Transition at Room Temperature. *Polyhedron* **2015**, *100*, 132–138. https://doi.org/10.1016/j.poly.2015.07.029

(5) Poodt, P. W. G.; Heijna, M. C. R.; Christianen, P. C. M.; van Enckevort, W. J. P.; de Grip, W. J.; Tsukamoto, K.; Maan, J. C.; Vlieg, E. Using Gradient Magnetic Fields to Suppress Convection during Crystal Growth. *Cryst Growth Des* **2006**, *6* (10), 2275–2280. https://doi.org/10.1021/cg0600546

(6) Poodt, P. W. G.; Christianen, P. C. M.; Enckevort, W. J. P. van; Maan, J. C.; Vlieg, E. The Critical Rayleigh Number in Low Gravity Crystal Growth from Solution. *Cryst Growth Des* **2008**, *8* (7), 2194–2199. https://doi.org/10.1021/cg070292g

(7) Wilcox, W. R. Transport Phenomena in Crystal Growth from Solution. *Prog Cryst Growth Charact Mater* **1993**, *26*, 153–194. https://doi.org/10.1016/0960-8974(93)90014-U

(8) Redhammer, G. J.; Koll, L.; Bernroider, M.; Tippelt, G.; Amthauer, G.; Roth, G. Co^{2+} - Cu^{2+} Substitution in Bieberite Solid-Solution Series, $(Co_{1-x}Cu_x)SO_{(4)}$(.)$7H_{(2)}O$,0.00. *Am Mineral* **2007**, *92* (4), 532–545. https://doi.org/10.2138/am.2007.2229
